# Common BACE2 Polymorphisms are Associated with Altered Risk for Alzheimer’s Disease and CSF Amyloid Biomarkers in APOE ε4 Non-Carriers

**DOI:** 10.1038/s41598-019-45896-4

**Published:** 2019-07-03

**Authors:** Matt Huentelman, Matthew De Both, Wayne Jepsen, Ignazio S. Piras, Joshua S. Talboom, Mari Willeman, Eric M. Reiman, John Hardy, Amanda J. Myers

**Affiliations:** 1 Neurogenomics Division, The Translational Genomics Research Institute (TGen), Phoenix, AZ USA; 2 The Arizona Alzheimer’s Consortium, Phoenix, AZ USA; 3 Banner Alzheimer’s Institute, Phoenix, AZ USA; 4 Neurodegenerative Disease Research Center, BioDesign Institute, Arizona State University, Tempe, AZ USA; 5 Department of Psychiatry, University of Arizona, Tucson, AZ USA; 6 Department of Molecular Neuroscience, Reta Lilla Weston Laboratories, and UK Dementia Research Institute at University College London, Institute of Neurology, London, United Kingdom; 7 Department of Psychiatry & Behavioral Sciences, Programs in Neuroscience and Human Genetics and Genomics and Center on Aging, Miller School of Medicine, University of Miami, Miami, FL USA

**Keywords:** Genetic predisposition to disease, Alzheimer's disease

## Abstract

It was recently suggested that beta-site amyloid precursor protein (APP)-cleaving enzyme 2 (BACE2) functions as an amyloid beta (Aβ)-degrading enzyme; in addition to its better understood role as an APP secretase. Due to this finding we sought to understand the possible genetic risk contributed by the *BACE2* locus to the development of late-onset Alzheimer’s disease (AD). In this study, we report that common single nucleotide polymorphism (SNP) variation in *BACE2* is associated with altered AD risk in apolipoprotein E gene (APOE) epsilon 4 variant (ε4) non-carriers. In addition, in ε4 non-carriers diagnosed with AD or mild cognitive impairment (MCI), SNPs within the *BACE2* locus are associated with cerebrospinal fluid (CSF) levels of Aβ1-42. Further, SNP variants in *BACE2* are also associated with BACE2 RNA expression levels suggesting a potential mechanism for the CSF Aβ1-42 findings. Lastly, overexpression of BACE2 *in vitro* resulted in decreased Aβ1-40 and Aβ1-42 fragments in a cell line model of Aβ production. These findings suggest that genetic variation at the *BACE2* locus modifies AD risk for those individuals who don’t carry the ε4 variant of APOE. Further, our data indicate that the biological mechanism associated with this altered risk is linked to amyloid generation or clearance possibly through BACE2 expression changes.

## Introduction

The amyloid (Aβ) hypothesis of Alzheimer’s disease (AD) has primarily been driven by the observation that genetic variability that alters amyloid precursor protein (APP) metabolism increases de position of Aβ^1^. Classically, the causative mutations were in *APP* itself and in the presenilin genes – PSEN1 and 2. Aβ is generated by the sequential actions of β-secretase (BACE1) and γ-secretase, of which the presenilins are the major component^2^. More recently, reduced function variants of both ADAM10^3^ and ADAM17^4^, the enzymes involved in the alternate α-secretase cleavage of APP, have been shown to increase the risk of AD as well. Finally, it is clear that the homologue of BACE1, BACE2 can also cleave both APP and amyloid beta (Aβ)^5,6^.

The actions of BACE2 may be involved in altering pathogenic Aβ fragment concentrations in multiple ways. It is known that BACE2 can cleave APP at the β-secretase site (albeit with much lower efficiency than BACE1) and near the α-secretase site as well^7–9^. Fluhrer *et al*. demonstrated that the cleavage of APP via BACE2 results in production of soluble APP-α and reduced production of amyloidogenic species suggesting that APP metabolism by BACE2 acts in an opposing fashion to BACE1 in cells that express both enzymes^8^. However, more recent research suggests that the primary activity of BACE2 occurs at a “θ-secretase” site (Phe^10^) located within the Aβ domain in APP. In 2006, Sun *et al*. identified this novel θ-secretase site and demonstrated that it results in the prevention of Aβ production^5^. Lastly, Abdul-Hay *et al*. demonstrated that BACE2 can degrade Aβ with an efficiency second only to insulin degrading enzyme (IDE)^6^.

Although two studies, one in the Finnish population^11^ and one in Down syndrome, have both implicated BACE2 in disease, to date neither exome sequencing or genome-wide association studies have reported common or rare pathogenic variability in either BACE1 or BACE2 as altering risk for disease. However, Wang *et al*. recently reported that mutations in the juxtamembrane helix (JH) domain of BACE2 results in a “conditional” β-secretase activity that leads to elevated Aβ production thereby establishing a potential genetic basis for BACE2 variation in AD risk^12^.

With this in mind we examined the *BACE2* locus, using a clinically characterized and neuropathology confirmed sample set (TGenII) as our starting point. We have previously shown that these confirmed samples increase the power to detect genetic associations. This is presumably because they avoid the misclassification of controls and the misdiagnosis of cases^13^. Next, we attempted to replicate findings in the Alzheimer’s Disease Neuroimaging Initiative (ADNI) cohort by examining the cerebrospinal fluid (CSF) biomarker data. Additionally, we examined the BACE2 locus for an expression quantitative trait (eQTL) to determine if variants in the region could be associated with BACE2 expression levels. Lastly, we confirmed that alteration of BACE2 expression influenced Aβ levels *in vitro*.

## Methods

### “TGenII” clinically characterized and neuropathologically verified cohort

Subjects from the TGenII neuropath cohort were collected as previously described^14^. We selected cases and controls as subjects of European ancestry who were verified pathologically as having AD or not, respectively. Subjects were greater than age 65 at death. Board-certified neuropathologists performed Braak staging based on the degree of neurofibrillary tangles^15^, and/or CERAD^16^ classification on the extent of neuritic plaques. Any samples with a history of other known neurological disease and or clinical history of stroke, cerebrovascular disease, and/or Lewy bodies were excluded.

### ADNI subjects

Subjects from the ADNI cohort (adni.loni.usc.edu) were collected as described previously^17^. Study participants were given both clinical and cognitive assessments. The mini-mental state exam (MMSE)^18^ was used to screen for and score cognitive impairment along with the clinical dementia rating (CDR)^19^ to determine level of dementia. AD subjects were determined as clinically according to the National Institute of Neurological and Communicative Disorders and Stroke and the Alzheimer’s Disease and Related Disorders Association (NINCDS-ADRDA) criteria^20^ and having MMSE scores between 20–26 and CDR between 0.5 and 1.0. Mild cognitive impairment (MCI) subjects had MMSE scores between 24–30, CDR = 0.5, reported memory complaints but no other signs of impairment of other cognitive functions, and were non-demented. Healthy normal controls (NC) had MMSE of 24–30, CDR = 0, and showed no signs of dementia. The primary goal of ADNI has been to test whether serial magnetic resonance imaging (MRI), positron emission tomography (PET), other biological markers, and clinical and neuropsychological assessment can be combined to measure the progression of mild cognitive impairment (MCI) and early Alzheimer’s disease (AD). For up-to-date information, see www.adni-info.org.

### Single nucleotide polymorphism (SNP) genotyping

The TGenII cohort was genotyped on the Affymetrix Genome-Wide Human SNP 6.0 Array (Affymetrix, Inc., Santa Clara, CA, USA) with standard manufacturer’s protocols (Affymetrix Genome-Wide Human SNP Nsp/Sty 6.0 User Guide; Rev. 1, 2007). The ADNI cohort was genotyped on the Illumina Human610-Quad BeadChip (Illumina, Inc. San Diego, CA) according to the manufacturer’s protocols (Infinium HD Assay; Super Protocol Guide; Rev. A, May 2008). Both SNP datasets are free and publicly available either via the main study sites or through the Alzheimer’s Disease Genetic Consortium (ADGC or https://www.nigads.org/datasets/ng00028).

### BACE2 locus imputation

Imputation was conducted with IMPUTE2 software (v2.1.2) using the multi-population reference panel approach^10,21^. We used the June 2011 release of the 1000 Genomes (1000genomes.org) Phase 1. We imputed a 5 megabase region encompassing *BACE2* on chromosome 21 between positions 40,000,000-45,000,000 in hg19 coordinates. For the phasing stage we used 80 haplotypes (-k option) as a template. For both the phasing and imputation rounds of IMPUTE2, we performed 10 burn in iterations (-burnin option) and performed 30 Markov chain Monte Carlo (MCMC) iterations (-iter option). Since we performed the multi-population approach, we set the “effective size” option (-Ne) to the suggested value of 20000. In addition, we utilized the strand alignment procedure (-fix_strand_g option) to minimize genotype strand discrepancies between the reference and study panels. GTOOL was used to convert IMPUTE2 GEN output to PED format.

### SNP and haplotype association testing

SNPs with minor allele frequencies less than 1% or with genotyping rate below 95% were filtered prior to analysis. For the initial hypothesis testing of BACE1 and BACE2 a Fisher’s exact test was utilized and 1000 max(T) permutations in PLINK^22^ were performed. After determining BACE2 warranted further follow-up, we assessed population structure and incorporated the results as covariates in regression models. We used ADMIXTURE^23^ (v1.04) with K = 3 by selecting a subset of directly genotyped SNPs (i.e., not imputed) with >99% call rates, minor allele frequency >0.3, pairwise R2 < 0.01. Q1 and Q2 vector solutions and sex were included as covariates in the regressions, and APOE ε4 carrier status was included as a covariate and genotype interaction term. Haplotypes were called with PLINK using the default parameters. Correction for multiple testing during SNP analysis was conducted using the Bonferroni method with independent SNPs considered to be those with r^2^ < 0.80.

### Aβ CSF measures

Aβ CSF levels were used from the ADNI dataset. The approach for CSF collection and measurement of the Aβ biomarker was reported previously^17^. The approach for testing Aβ1-42 fragment association with BACE2 was carried out in a similar fashion to the AD association; however, instead of using case/control as binary phenotypes, we performed a linear regression with Aβ1-42 levels as the phenotype. The imputed BACE2 SNPs were tested against Aβ1-42 CSF measures with sex and ADMIXTURE K = 3 Q1 and Q2 values as covariates.

### *In vitro* Aβ ELISA measurements

BACE2 overexpression was achieved using the Sleeping Beauty Transposon system^24^. BE(2)-m17 neuroblastoma cells were plated in uncoated, 6-well plates (14e6 cells/well) and media exchanged every 24 hours. Seventy-two-hours after plating-, supernatant was collected and supplemented with 4-(2-aminoethyl)benzenesulfonyl fluoride hydrochloride (AEBSF) to a final concentration of 1.0 mM. Samples were clarified, decanted, and stored at −80 °C until analysis. Extracellular Aβ levels were quantified using the Human βAmyloid [1–40] (ThermoFisher Scientific) and the Human Aβ [1-42] ELISAs (ThermoFisher Scientific), according to the manufacturer’s instructions.

### Highlights

- SNPs within the *BACE2* locus were associated with altered Alzheimer’s disease risk preferentially in APOE ε4 non-carriers.
- In individuals with diagnosed AD or MCI who do not carry an APOE ε4 allele, SNPs within *BACE2* were associated with differential Aβ1-42 levels in the CSF.
- SNPs within *BACE2* are associated with *BACE2* mRNA levels in control individuals via eQTL analysis and this effect is modified by APOE status.
- Overexpression of BACE2 in a cell line that naturally produces Aβ peptides results in lower amounts of Aβ1-40 and Aβ1-42.
- Considered together, these data suggest that genetic variation at the *BACE2* locus is associated with AD risk and that it may be functionally linked to altered Aβ processing.

## Results and Discussion

**Figure 1:**
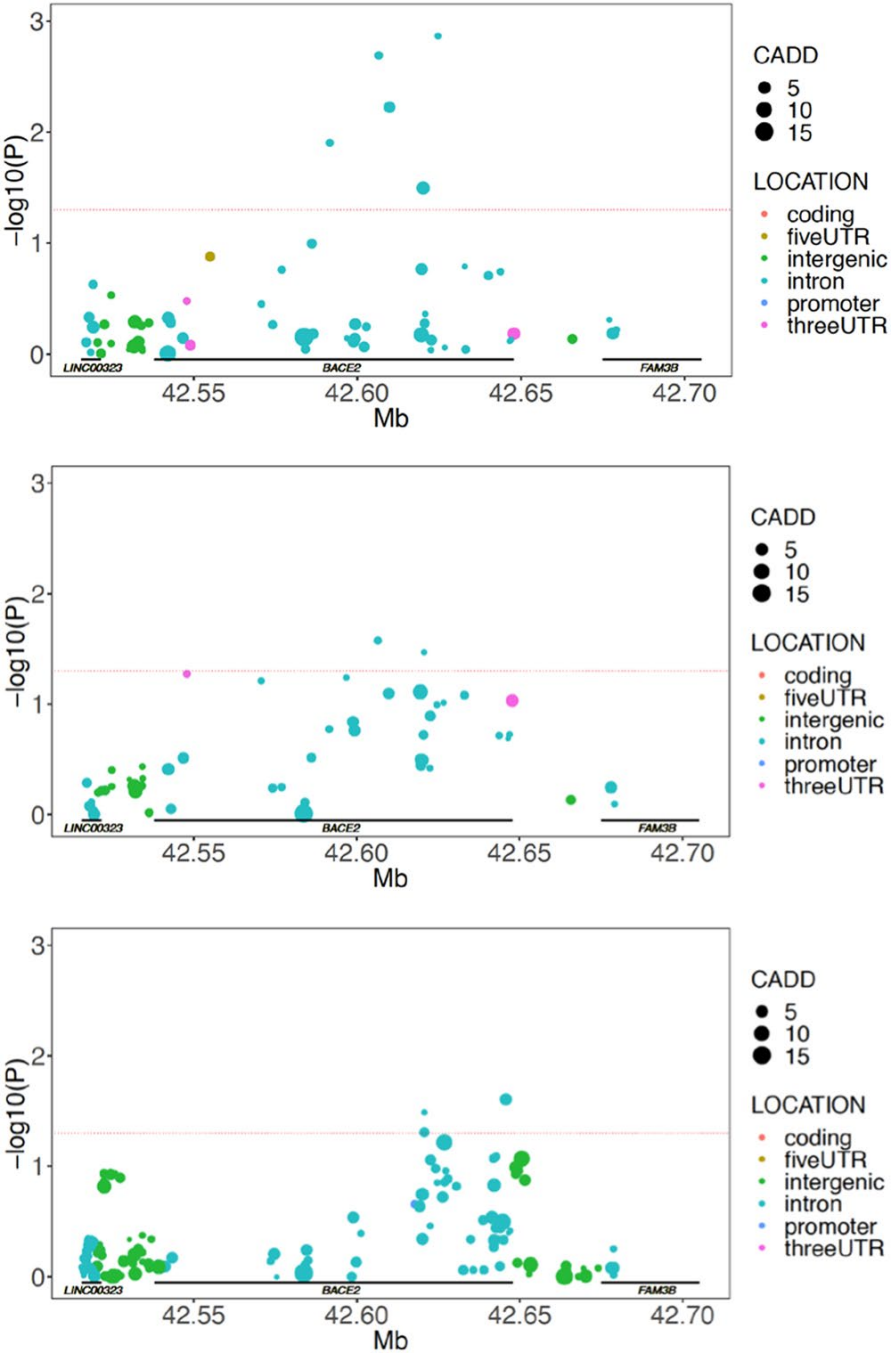
AD risk is associated with SNPs within the BACE2 locus in APOE ε4 non-carriers. BACE2 association in the clinically characterized and neuropathologically verified cohort. In all panels, SNP uncorrected P-values are plotted as colored circles based on their location within the genetic locus, the size of the circle is representative of the CADD score for the SNP. A horizontal red dashed line represents nominal significance of p = 0.05. (Top panel) BACE2 association in all discovery subjects (N = 1599; 1014 cases; 585 controls). (Middle) BACE2 interaction p-values with APOE carrier status (Bottom) BACE2 interaction p-values with APOE carrier status including the first two population structure vectors and sex.

We initiated our study in a cohort of clinically characterized and neuropathologically verified AD cases and controls (n = 1599; 1014 cases, 585 controls) known as “TGenII”. This cohort is advantageous for genetic study over living, clinically diagnosed AD cohorts due to the absence of the mis-assignment of cases and controls. We examined the BACE2 locus in three steps. First, we examined the association at the locus using a simple logistic regression case:control analysis examining genotyped and imputed SNPs (Fig. 1, top panel). The SNP rs8134992 was identified as the most significant with an uncorrected p-value of 0.00136. Next, we examined this association after including APOE carrier status (e.g. 0, 1, or 2 APOE ε4 alleles) as an interaction term in the additive logistic regression model (Fig. 1, middle panel). This yielded a significant association at rs2898441 with an uncorrected p-value of 0.0267. In a final analysis of this association we included the first two population structure co-variates and sex as interaction terms in addition to APOE carrier status (Fig. 1, bottom panel). This resulted in rs9978431 as the SNP with the most significant p-value at 0.0249. All of the data for these comparisons can be found in Supplemental Tables 1–3. The interaction with APOE status was further analyzed by performing a post-hoc examination of the association signal across the locus by splitting the cohort into APOE carriers and non-carriers. This demonstrated that the association within *BACE2* was only significant in the APOE ε4 non-carriers (Supplemental Fig. 1). In an attempt to relate potential functional impact to the SNPs we utilized CADD scoring^25^. There were no SNPs with CADD scores over 15 suggesting that the associated SNPs do not have direct impact at the protein function level.

These association results, in a well characterized neuropathology-confirmed cohort, suggest that there are common SNPs within the *BACE2* locus that alter AD risk preferentially in individuals who do not carry the APOE ε4 genetic risk for the disease. This is important to note due to the role of BACE2 in amyloid processing. Due to this finding, we next hypothesized that genetic variation at *BACE2* would be associated with Aβ1-42 biomarker levels due to its ability to degrade the peptide or via its theta-secretase actions which could lead to lowered Aβ1-42 production by shunting APP metabolism down a non-amyloidogenic path.

**Figure 2:**
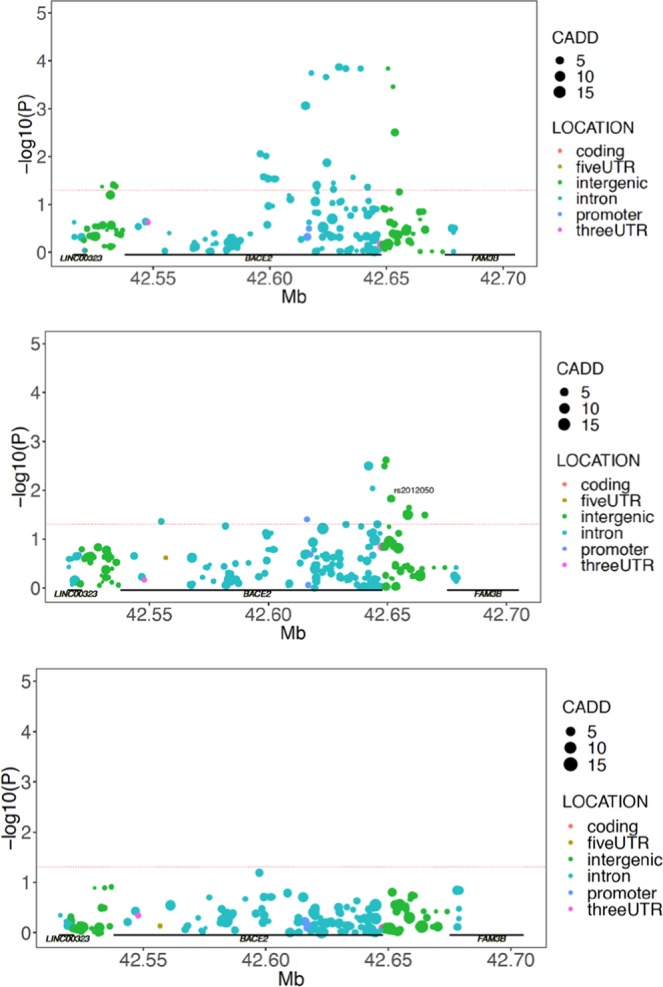
CSF Aβ1-42 levels are associated with BACE2 locus SNPs in AD and MCI Individuals from the ADNI cohort who are APOE ε4 non-carriers. BACE2 association with Aβ1-42 CSF levels in ADNI cohort AD, MCI, and control NA individuals including APOE carrier status, the first two population structure vectors, and sex as interaction terms. In all panels, uncorrected SNP P-values are plotted as colored circles based on their location within the genetic locus, the size of the circle is representative of the CADD score for the SNP. A horizontal red dashed line represents nominal significance of p = 0.05. (Top panel) BACE2 interaction p-values in ADNI AD individuals. (Middle) BACE2 interaction p-values in ADNI MCI individuals. (Bottom) BACE2 interaction p-values in ADNI control NA individuals.

Using the ADNI cohort and a linear regression analysis incorporating the first two population structure co-variates, sex, and APOE carrier status as interaction terms, we demonstrated an association with CSF Aβ 1-42 levels that was significant in the MCI and AD individuals (Fig. 2). The strongest associated SNPs (rs2837994 in AD at p = 0.000183 and rs9980146 in MCI at p = 0.0207, both uncorrected) were located in the same region as defined above for the BACE2 genetic association. Of note, there was no significant association in the NA control individuals in ADNI suggesting that this locus may be preferentially relevant for CSF Aβ1-42 biomarker levels during disease processes. As before, we utilized a post-hoc analysis to identify which APOE group was associated with the CSF Aβ1-42 changes and found that the association was again only significant in APOE ε4 non-carriers. This is in agreement with the genetic associations we detected in the first phase of our study. The full results of this analysis can be found in Supplemental Tables 4–6.

The work in the ADNI cohort further supports the association at the *BACE2* locus and its specificity in APOE ε4 non-carriers. No variants identified during the CADD scoring approach clearly have the potential to alter protein function. To that end, we predicted that BACE2 gene expression levels may be altered by the associated variants; thus, in turn, altered levels of BACE2 mRNA and protein would lead to an alteration in Aβ1-42.

**Figure 3:**
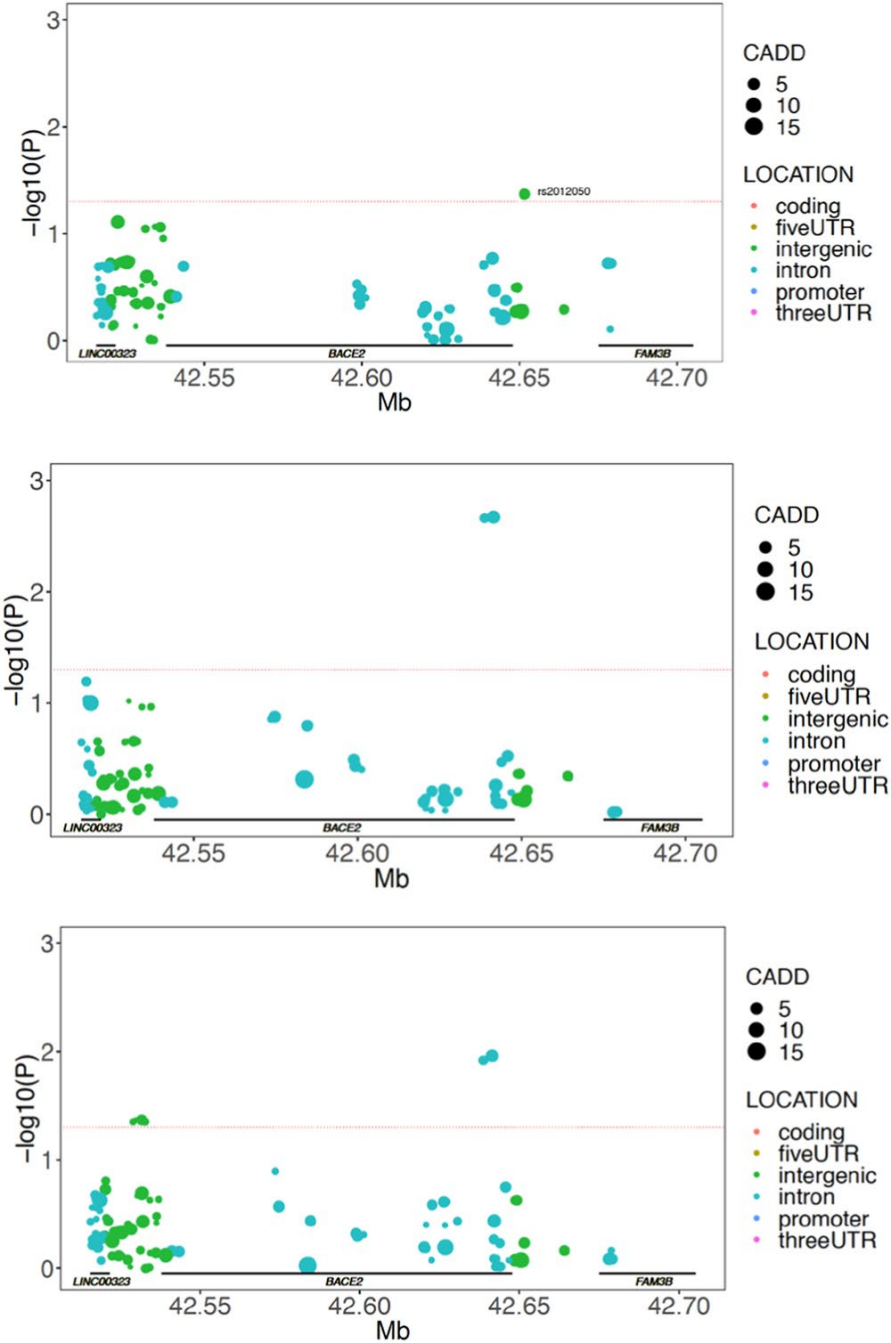
BACE2 locus SNPs are associated with BACE2 gene expression levels in cortex samples from control donors. eQTL p-values are indicated for BACE2 SNPs across three different array probes for BACE2 expression. In all panels, uncorrected SNP P-values are plotted as colored circles based on their location within the genetic locus, the size of the circle is representative of the CADD score for the SNP. A horizontal red dashed line represents nominal significance of p = 0.05. (Top panel) BACE2 eQTL p-values in control individuals for probe GI21040361-A. (Middle) BACE2 eQTL p-values in control individuals for probe ILMN1669323. (Bottom) BACE2 eQTL p-values in control individuals for probe ILMN2326712.

**Figure 4:**
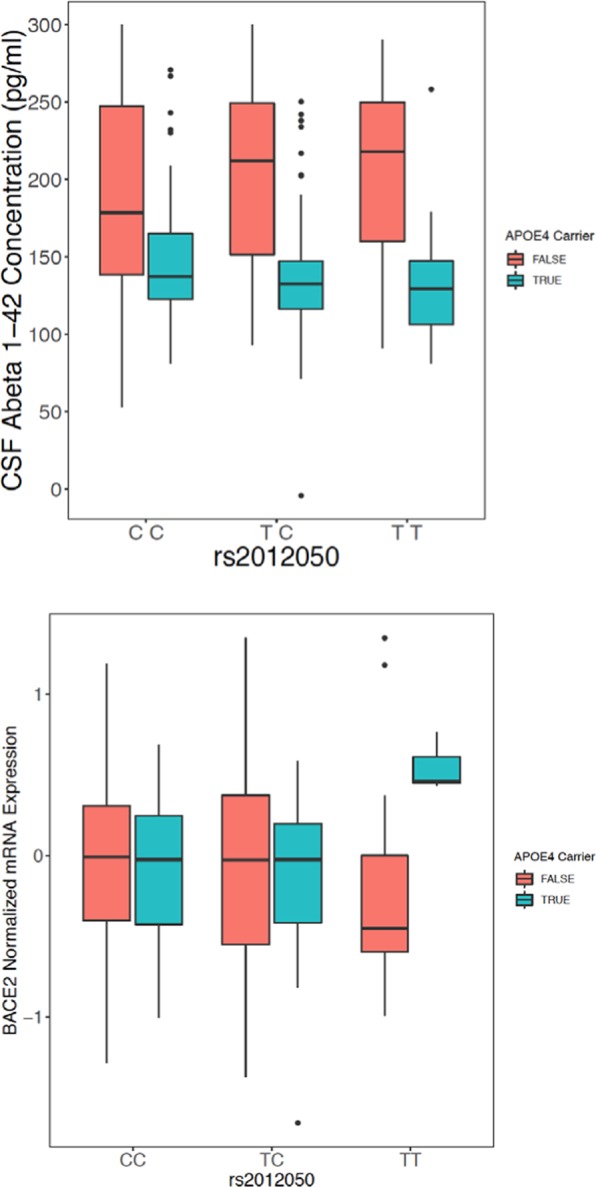
SNP rs2012050 is associated with altered BACE2 gene expression and Aβ1-42 CSF levels. The relationship between rs2012050 genotype (x-axis) and either Aβ1-42 CSF levels (Top panel) or BACE2 gene expression (Bottom) is shown. APOE carrier status is indicated by the color of the boxplot; red = APOE ε4 non-carriers, blue = APOE ε4 carriers. Note the higher levels of Aβ1-42 in the CSF from rs2012050 T/T individuals who are APOE ε4 non-carriers as well as a lower expression of the BACE2 gene in individuals with the same genetic characteristics.

To examine this we utilized an expression quantitative trait locus (eQTL) analysis approach to identify variants within the *BACE2* locus that may be associated with *BACE2* RNA levels. We utilized array-based gene expression data from the TGenII cohort cortical brain tissue samples from control individuals only (to avoid any confounding influence of disease on gene expression). Three probes that are specific to BACE2 were examined and all three demonstrated an eQTL association that was modified by APOE carrier status (Fig. 3). Of particular note was SNP rs2012050 (p = 0.0425, uncorrected) which was also identified as significant in the ADNI MCI cohort CSF Aβ1-42 analysis. APOE ε4 non-carriers of the minor allele of rs2012050 demonstrate lower BACE2 gene expression and also higher CSF Aβ1-42 levels (Fig. 4). This is in agreement with the potential actions of BACE2 on Aβ1-42. All of the eQTL data can be found in Supplemental Tables 7–9.

**Figure 5:**
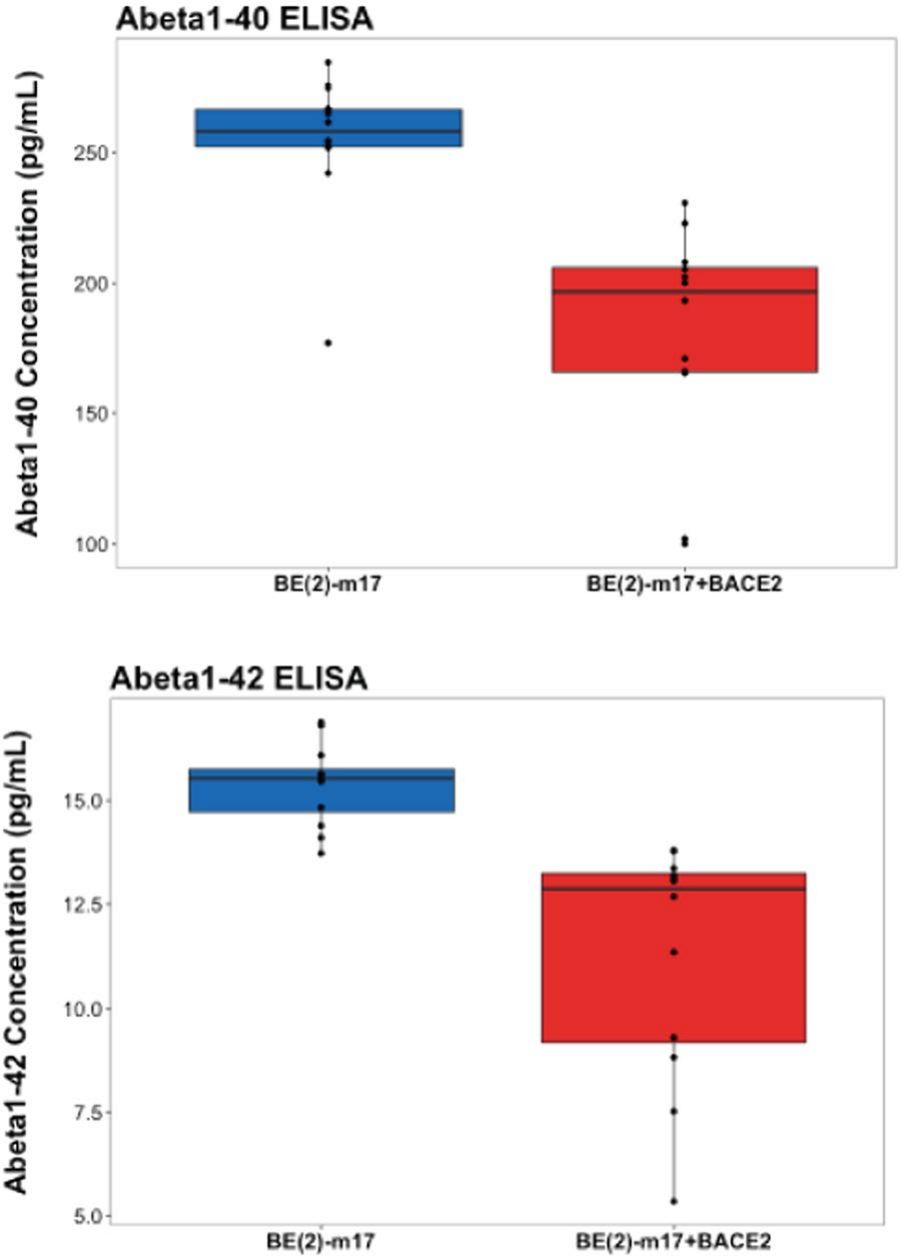
BACE2 overexpression results in lower levels of Aβ 1-40 and 1–42 peptides in cell culture supernatant from BE(2)-m17 cells. AB1-40 and 1–42 levels were measured in the cell culture supernatant from wild-type (blue box plots) or cells with BACE2 overexpression (red box plots).

Next we overexpressed BACE2 in a cell line that we showed previously expresses the necessary genetic components and produces Aβ fragments *in vitro*^26^. Notably, this cell line expresses BACE2 at very low levels (less than 0.5 FPKM based on RNA sequencing data, not shown). We overexpressed BACE2 in this cell line and demonstrated that this resulted in lower amounts of Aβ1-40 and 1-42 fragments in the cell culture media (Fig. 5). This final experiment confirmed other reports in the literature demonstrating that BACE2 can degrade Aβ peptides^6^. Further, these data suggest that the alteration of BACE2 expression may result in the CSF level alterations we observed.

Collectively, this work supports previous analyses suggesting that genetic variability in BACE2 alters mRNA expression and plays a role in AD. Understanding the precise role of BACE2 in APP/Aβ metabolism is clearly important because drugs designed as BACE1 inhibitors also demonstrate cross-reactivity with BACE2. The effect of BACE2 inhibition on APP processing would likely be anti-amyloidogenic since it would lead to less Aβ being formed^8^, but inhibition of BACE2 may also reduce its cleavage of Aβ. However, it is noteworthy that Sun *et al*.^5^ suggested increasing BACE2 activity was a valid approach for AD therapy given its high propensity to degrade Aβ. This suggests that inhibition of BACE2 is not a desirable approach. A further complication, when trying to predict the effects of BACE2 inhibition on disease etiology, is that BACE2 expression is high in both neurons and glial cells. This means that with the gliosis inherent in the AD disease process, BACE2 expression may become more pronounced as the disease progresses. Indeed, it is possible that such a BACE2 effect on pathogenesis during AD disease progression may underlie the APOE effect we report herein. Importantly, our data suggest that BACE2 has important roles in APP/Aβ metabolism. Indeed, this has wide reaching implications 1) for assessment of BACE inhibition in AD treatment, and 2) in the interpretation of AD rodent models where the Swedish (“BACE1 preferring”) mutation is used to model the disease.

## Supplementary information

Supplementary Information

## Data Availability

All SNP genotypes and CSF biomarker levels are available openly via the respective study sites or through the ADGC.
