# Supplementary material for: Common BACE2 Polymorphisms are Associated with Altered Risk for Alzheimer’s Disease and CSF Amyloid Biomarkers in APOE ε4 Non-Carriers
Source: Sci Rep. 2019 Jul 3;9:9640. doi: 10.1038/s41598-019-45896-4 (PMC6610620; doi:10.1038/s41598-019-45896-4)
Supplement: Supplementary file 1 — Supplementary Information [file 41598_2019_45896_MOESM1_ESM.pdf]

Supplementary Information

Common BACE2 Polymorphisms are Associated with Altered Risk for Alzheimer's Disease and CSF Amyloid Biomarkers in APOE ε4 Non-Carriers

Matt Huentelman[a,b,§,*] Matthew DeBoth[a,b], Wayne Jepsen[a,b], Ignazio S. Piras[a,b], Joshua S. Talboom[a,b], Mari Willeman[a,b], Eric M. Reiman[b,c,d,e], John Hardy[f], and Amanda J. Myers[g]

[a]Neurogenomics Division, The Translational Genomics Research Institute (TGen), Phoenix, AZ USA

[b]The Arizona Alzheimer's Consortium, Phoenix, AZ, USA

[c]Banner Alzheimer's Institute, Phoenix, AZ, USA

[d]Neurodegenerative Disease Research Center, BioDesign Institute, Arizona State University, Tempe, AZ, USA

[e]Department of Psychiatry, University of Arizona, USA

[f]Department of Molecular Neuroscience and Reta Lilla Weston Laboratories, Institute of Neurology, London, United Kingdom

[g]Department of Psychiatry & Behavioral Sciences, Programs in Neuroscience and Human Genetics and Genomics and Center on Aging, Miller School of Medicine, University of Miami, Miami, FL USA

SUPPLEMENTAL FIGURE and TABLE LEGENDS

Supplemental Figure 1. AD risk is associated with SNPs within the BACE2 locus in APOE ε4 non-carriers.

In both panels, uncorrected SNP P-values are plotted as colored circles based on their location within the genetic locus, the size of the circle is representative of the CADD score for the SNP. A horizontal red dashed line represents nominal significance of p=0.05. (Top panel) BACE2 association in only the APOE ε4 carrying individuals. (Bottom) BACE2 association in only the APOE ε4 non-carrying individuals. Note the presence of significant association in only the APOE ε4 non-carrying cohort.

Supplemental Table 1. Complete table for the data represented in Figure 1, Top

Supplemental Table 2. Complete table for the data represented in Figure 1, Middle

Supplemental Table 3. Complete table for the data represented in Figure 1, Bottom

Supplemental Table 4. Complete table for the data represented in Figure 2, Top

Supplemental Table 5. Complete table for the data represented in Figure 2, Middle

Supplemental Table 6. Complete table for the data represented in Figure 2, Bottom

Supplemental Table 7. Complete table for the data represented in Figure 3, Top

Supplemental Table 8. Complete table for the data represented in Figure 3, Middle

Supplemental Table 9. Complete table for the data represented in Figure 3, Bottom

Supplementary Figure 1

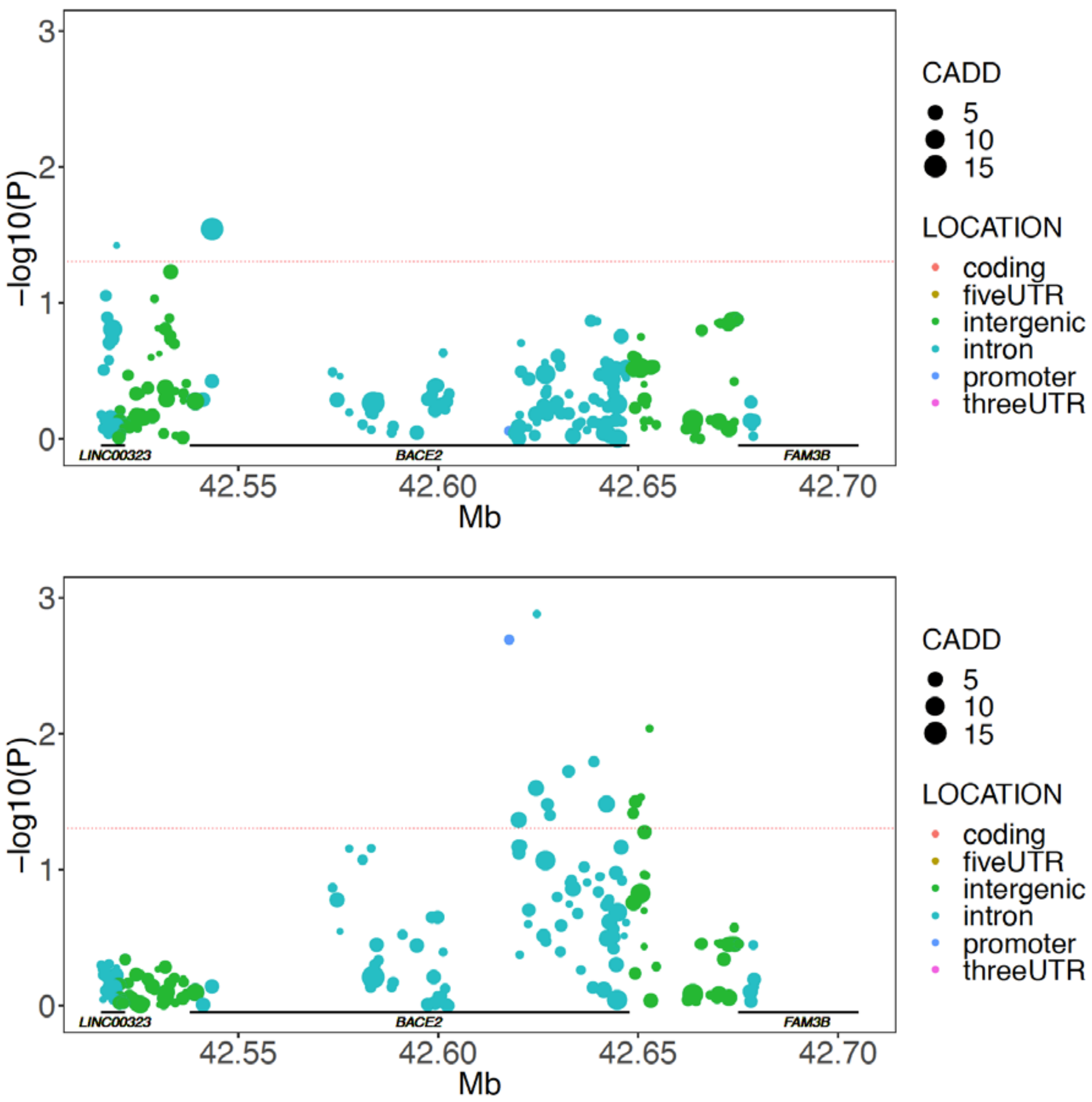

Supplementary Table 1

CHR	SNP	BP	A1	TEST	NMISS	OR	SE	L95	U95	STAT	P
21	rs8134992	42624622	T	ADD	1597	0.7071	0.1082	0.5719	0.8742	-3.202	0.001364
21	rs2898441	42606524	C	ADD	1596	0.7968	0.07366	0.6897	0.9206	-3.083	0.002046
21	rs2837977	42609869	A	ADD	1546	0.8126	0.07547	0.7009	0.9422	-2.749	0.005981
21	rs2837970	42591611	T	ADD	1582	0.8221	0.07832	0.7051	0.9586	-2.5	0.01241
21	rs914187	42619749	A	ADD	1554	1.179	0.07638	1.015	1.369	2.151	0.03151
21	rs2837989	42620119	A	ADD	1588	0.8209	0.09195	0.6855	0.983	-2.146	0.03186
21	rs9983496	42586130	C	ADD	1591	0.8812	0.07713	0.7575	1.025	-1.64	0.1009
21	rs7278659	42555005	A	ADD	1545	1.367	0.2082	0.9089	2.056	1.501	0.1333
21	rs11701756	42632770	T	ADD	1593	1.24	0.1534	0.918	1.675	1.402	0.1609
21	rs914186	42619657	G	ADD	1592	1.114	0.07871	0.9547	1.3	1.371	0.1704
21	rs2837967	42576964	A	ADD	1591	0.8857	0.0889	0.7441	1.054	-1.365	0.1722
21	rs4818227	42643729	T	ADD	1538	0.8946	0.08306	0.7602	1.053	-1.342	0.1797
21	rs3787945	42640022	T	ADD	1591	1.223	0.1559	0.9009	1.66	1.29	0.1969
21	rs2837963	42519356	C	ADD	1596	1.097	0.07794	0.9418	1.278	1.191	0.2338
21	rs2251871	42524842	C	ADD	1588	1.084	0.07683	0.9322	1.26	1.047	0.2952
21	rs4818219	42547894	C	ADD	1589	1.081	0.08021	0.9239	1.265	0.9732	0.3305
21	rs2837966	42570701	C	ADD	1564	0.913	0.09827	0.753	1.107	-0.9266	0.3541
21	rs2837993	42620714	A	ADD	1594	1.06	0.07519	0.9152	1.229	0.7809	0.4349
21	rs1571733	42518034	T	ADD	1595	0.9273	0.1034	0.7571	1.136	-0.7299	0.4654
21	rs2252503	42542204	A	ADD	1575	1.056	0.07541	0.9108	1.224	0.7208	0.471
21	rs928300	42676917	A	ADD	1578	0.8806	0.185	0.6128	1.266	-0.6872	0.492
21	rs7276900	42532075	A	ADD	1581	0.948	0.08111	0.8087	1.111	-0.6577	0.5107
21	rs2776344	42542987	G	ADD	1594	0.9486	0.0817	0.8083	1.113	-0.6457	0.5185
21	rs2210277	42536308	C	ADD	1595	0.9493	0.08069	0.8105	1.112	-0.6443	0.5194
21	rs6517655	42536246	G	ADD	1571	0.9494	0.08133	0.8095	1.113	-0.6382	0.5234
21	rs2837992	42620520	A	ADD	1598	1.049	0.07638	0.9035	1.219	0.6314	0.5278
21	rs1077117	42599364	G	ADD	1595	0.9476	0.08672	0.7995	1.123	-0.6204	0.535
21	rs11701197	42522788	A	ADD	1550	1.057	0.09061	0.8851	1.262	0.6124	0.5403
21	rs4816713	42574184	T	ADD	1597	0.9392	0.103	0.7676	1.149	-0.6092	0.5424
21	rs7279020	42534031	A	ADD	1576	0.9558	0.07547	0.8244	1.108	-0.5986	0.5495
21	rs2837974	42602735	G	ADD	1597	0.9315	0.1226	0.7326	1.185	-0.5785	0.563
21	rs9982271	42519389	A	ADD	1596	0.9517	0.08687	0.8027	1.128	-0.5693	0.5692
21	rs9976965	42679090	C	ADD	1589	0.9551	0.08724	0.805	1.133	-0.526	0.5989
21	rs2837968	42586604	T	ADD	1596	1.092	0.1895	0.7529	1.583	0.4626	0.6436
21	rs10775668	42678029	T	ADD	1594	0.9604	0.08788	0.8085	1.141	-0.4592	0.6461
21	rs12149	42647821	A	ADD	1597	1.034	0.07343	0.8951	1.194	0.4514	0.6517
21	rs914183	42586495	C	ADD	1595	1.057	0.1228	0.8308	1.344	0.4501	0.6527
21	rs2837988	42619544	A	ADD	1595	0.9679	0.07521	0.8353	1.122	-0.4331	0.6649
21	rs6517656	42583738	A	ADD	1595	1.04	0.1001	0.8546	1.265	0.3912	0.6956

21	rs766850	42546817	T	ADD	1591	0.9669	0.09241	0.8067	1.159	-0.3648	0.7153
21	rs12329755	42596837	G	ADD	1599	0.973	0.07642	0.8377	1.13	-0.3582	0.7202
21	rs17000688	42599218	T	ADD	1596	0.9572	0.1245	0.7499	1.222	-0.3513	0.7254
21	rs1888517	42665680	G	ADD	1597	1.025	0.07162	0.8911	1.18	0.3502	0.7262
21	rs960231	42622657	C	ADD	1581	1.026	0.07788	0.8808	1.195	0.3298	0.7415
21	rs2838001	42646992	C	ADD	1598	1.031	0.0913	0.8617	1.232	0.3292	0.742
21	rs9981685	42533108	G	ADD	1591	0.9662	0.1154	0.7706	1.211	-0.2982	0.7656
21	rs7278856	42598887	G	ADD	1580	0.9746	0.08701	0.8218	1.156	-0.2959	0.7673
21	rs2838000	42646518	A	ADD	1592	1.027	0.09181	0.8579	1.23	0.2909	0.7711
21	rs11700917	42517222	T	ADD	1597	1.028	0.09881	0.8472	1.248	0.2821	0.7779
21	rs2837964	42520646	A	ADD	1598	1.022	0.07992	0.8739	1.195	0.2738	0.7843
21	rs11702600	42524832	C	ADD	1586	1.023	0.08941	0.8587	1.219	0.2561	0.7978
21	rs9015	42549007	A	ADD	1598	1.066	0.2898	0.6041	1.881	0.2208	0.8252
21	rs2150425	42531759	T	ADD	1598	1.015	0.07667	0.8736	1.18	0.1967	0.8441
21	rs2837973	42602088	G	ADD	1592	0.9777	0.1243	0.7663	1.247	-0.1814	0.8561
21	rs2837996	42626706	T	ADD	1596	0.9877	0.07794	0.8477	1.151	-0.1593	0.8734
21	rs6517653	42534253	T	ADD	1595	0.9857	0.1008	0.809	1.201	-0.1427	0.8865
21	rs6517657	42584141	A	ADD	1589	1.013	0.1005	0.8317	1.233	0.1259	0.8998
21	rs4818226	42633065	T	ADD	1583	1.01	0.07887	0.865	1.178	0.1214	0.9034
21	rs2016240	42530260	T	ADD	1593	0.9891	0.1019	0.81	1.208	-0.1078	0.9142
21	rs960230	42622479	T	ADD	1596	1.009	0.09028	0.8455	1.204	0.1006	0.9199
21	rs6517654	42534328	C	ADD	1593	1.007	0.08852	0.847	1.198	0.0838	0.9332
21	rs2837961	42518599	G	ADD	1585	1.004	0.07316	0.8696	1.158	0.05086	0.9594
21	rs2297272	42542175	C	ADD	1573	0.9973	0.1076	0.8077	1.231	-0.02544	0.9797
21	rs9974272	42521711	C	ADD	1597	0.9986	0.08843	0.8397	1.188	-0.01551	0.9876

Supplementary Table 2

CHR	SNP	BP	A1	TEST	NMISS	OR	SE	L95	U95	STAT	P
21	rs2898441	42606524	C	ADDxapoe_carrier	1596	1.494	0.1813	1.047	2.132	2.215	0.02673
21	rs2837993	42620714	A	ADDxapoe_carrier	1594	0.683	0.18	0.48	0.9719	-2.118	0.03413
21	rs4818219	42547894	C	ADDxapoe_carrier	1589	1.791	0.3011	0.9926	3.231	1.935	0.05297
21	rs12329755	42596837	G	ADDxapoe_carrier	1599	1.519	0.2201	0.9867	2.338	1.899	0.05756
21	rs2837966	42570701	C	ADDxapoe_carrier	1564	0.5077	0.3628	0.2493	1.034	-1.868	0.0617
21	rs2837988	42619544	A	ADDxapoe_carrier	1595	1.448	0.2095	0.9604	2.184	1.767	0.0772
21	rs2837977	42609869	A	ADDxapoe_carrier	1546	1.381	0.1842	0.9625	1.981	1.753	0.07967
21	rs4818226	42633065	T	ADDxapoe_carrier	1583	1.547	0.2515	0.9447	2.532	1.734	0.08299
21	rs12149	42647821	A	ADDxapoe_carrier	1597	0.7384	0.1802	0.5186	1.051	-1.683	0.09237
21	rs2837996	42626706	T	ADDxapoe_carrier	1596	1.473	0.234	0.9311	2.33	1.655	0.09791
21	rs8134992	42624622	T	ADDxapoe_carrier	1597	2.435	0.5434	0.8395	7.064	1.638	0.1014
21	rs960231	42622657	C	ADDxapoe_carrier	1581	1.416	0.2285	0.905	2.216	1.523	0.1278
21	rs7278856	42598887	G	ADDxapoe_carrier	1580	1.569	0.3077	0.8584	2.868	1.464	0.1433
21	rs2837970	42591611	T	ADDxapoe_carrier	1582	1.332	0.208	0.8859	2.002	1.377	0.1684
21	rs1077117	42599364	G	ADDxapoe_carrier	1595	1.525	0.3088	0.8325	2.793	1.366	0.1719
21	rs2838001	42646992	C	ADDxapoe_carrier	1598	1.828	0.4592	0.7434	4.497	1.314	0.1888
21	rs2837992	42620520	A	ADDxapoe_carrier	1598	1.321	0.2126	0.8709	2.004	1.31	0.1902
21	rs4818227	42643729	T	ADDxapoe_carrier	1538	1.394	0.2551	0.8457	2.299	1.303	0.1925
21	rs2838000	42646518	A	ADDxapoe_carrier	1592	1.789	0.4613	0.7243	4.418	1.261	0.2074
21	rs9983496	42586130	C	ADDxapoe_carrier	1591	1.223	0.1955	0.8335	1.794	1.028	0.3038
21	rs766850	42546817	T	ADDxapoe_carrier	1591	1.469	0.3776	0.7011	3.08	1.019	0.308
21	rs914186	42619657	G	ADDxapoe_carrier	1592	1.254	0.2257	0.8059	1.952	1.004	0.3153
21	rs2837989	42620119	A	ADDxapoe_carrier	1588	1.433	0.3628	0.7038	2.918	0.9916	0.3214
21	rs914187	42619749	A	ADDxapoe_carrier	1554	1.202	0.2008	0.811	1.782	0.9168	0.3592
21	rs6517653	42534253	T	ADDxapoe_carrier	1595	1.663	0.5667	0.5478	5.05	0.898	0.3692
21	rs960230	42622479	T	ADDxapoe_carrier	1596	1.402	0.3871	0.6564	2.993	0.8725	0.3829
21	rs2252503	42542204	A	ADDxapoe_carrier	1575	0.8544	0.1819	0.5982	1.22	-0.8652	0.3869
21	rs2251871	42524842	C	ADDxapoe_carrier	1588	0.8527	0.1887	0.5891	1.234	-0.8445	0.3984
21	rs6517654	42534328	C	ADDxapoe_carrier	1593	0.8136	0.2872	0.4634	1.428	-0.7183	0.4726
21	rs2016240	42530260	T	ADDxapoe_carrier	1593	1.492	0.5702	0.4881	4.562	0.702	0.4827
21	rs11700917	42517222	T	ADDxapoe_carrier	1597	1.438	0.5594	0.4804	4.306	0.6497	0.5159
21	rs7279020	42534031	A	ADDxapoe_carrier	1576	0.8928	0.1863	0.6197	1.286	-0.6086	0.5428
21	rs2150425	42531759	T	ADDxapoe_carrier	1598	0.8917	0.1925	0.6114	1.3	-0.5956	0.5515
21	rs11702600	42524832	C	ADDxapoe_carrier	1586	0.8381	0.2984	0.467	1.504	-0.5917	0.554
21	rs2837967	42576964	A	ADDxapoe_carrier	1591	0.8385	0.3053	0.4609	1.525	-0.5771	0.5639
21	rs10775668	42678029	T	ADDxapoe_carrier	1594	0.8491	0.2859	0.4848	1.487	-0.572	0.5673
21	rs4816713	42574184	T	ADDxapoe_carrier	1597	1.405	0.6073	0.4272	4.618	0.5596	0.5758
21	rs11701197	42522788	A	ADDxapoe_carrier	1550	1.191	0.3375	0.6148	2.308	0.5182	0.6043
21	rs9974272	42521711	C	ADDxapoe_carrier	1597	0.8626	0.2898	0.4888	1.522	-0.5101	0.61
21	rs7276900	42532075	A	ADDxapoe_carrier	1581	0.891	0.2325	0.5649	1.405	-0.4965	0.6195

21	rs2837964	42520646	A	ADDxapoe_carrier	1598	1.127	0.2479	0.6933	1.832	0.4824	0.6295
21	rs1888517	42665680	G	ADDxapoe_carrier	1597	1.059	0.1717	0.7564	1.483	0.3342	0.7382
21	rs2837961	42518599	G	ADDxapoe_carrier	1585	0.9495	0.1734	0.6759	1.334	-0.2987	0.7652
21	rs6517657	42584141	A	ADDxapoe_carrier	1589	1.126	0.4143	0.5	2.537	0.2869	0.7742
21	rs9976965	42679090	C	ADDxapoe_carrier	1589	0.9317	0.2816	0.5365	1.618	-0.2511	0.8018
21	rs1571733	42518034	T	ADDxapoe_carrier	1595	0.9057	0.4642	0.3646	2.25	-0.2134	0.831
21	rs2776344	42542987	G	ADDxapoe_carrier	1594	1.034	0.2488	0.6351	1.684	0.135	0.8926
21	rs2837963	42519356	C	ADDxapoe_carrier	1596	0.9791	0.2089	0.6501	1.475	-0.1011	0.9195
21	rs2210277	42536308	C	ADDxapoe_carrier	1595	0.9868	0.2367	0.6205	1.569	-0.05616	0.9552
21	rs6517656	42583738	A	ADDxapoe_carrier	1595	1.015	0.3922	0.4706	2.189	0.03796	0.9697
21	rs9982271	42519389	A	ADDxapoe_carrier	1596	1.005	0.2918	0.5675	1.781	0.01861	0.9852
21	rs6517655	42536246	G	ADDxapoe_carrier	1571	1.001	0.2389	0.6266	1.599	0.003593	0.9971

Supplementary Table 3

CHR	SNP	BP	A1	TEST	NMISS	OR	SE	L95	U95	STAT	P
21	rs9978431	42645737	T	ADDxapoe_carrier	1554	1.512	0.1844	1.054	2.171	2.244	0.02485
21	rs2837993	42620714	T	ADDxapoe_carrier	1562	0.6869	0.1756	0.4869	0.969	-2.139	0.03242
21	rs9981553	42620680	T	ADDxapoe_carrier	1592	0.71	0.1738	0.505	0.9982	-1.97	0.04882
21	rs12482462	42626851	T	ADDxapoe_carrier	1569	0.7205	0.1749	0.5114	1.015	-1.875	0.06083
21	rs2837996	42626706	T	ADDxapoe_carrier	1528	1.543	0.2336	0.976	2.438	1.856	0.06347
21	rs9305733	42642699	C	ADDxapoe_carrier	1574	0.7394	0.1733	0.5264	1.038	-1.742	0.08144
21	rs2838004	42650654	G	ADDxapoe_carrier	1590	0.7434	0.1722	0.5304	1.042	-1.722	0.08508
21	rs9976426	42641913	T	ADDxapoe_carrier	1573	0.7422	0.1733	0.5284	1.042	-1.72	0.0854
21	rs960231	42622657	G	ADDxapoe_carrier	1563	1.466	0.224	0.9453	2.275	1.709	0.0875
21	rs12482242	42648821	G	ADDxapoe_carrier	1591	0.7545	0.1721	0.5385	1.057	-1.636	0.1017
21	rs2837994	42624124	A	ADDxapoe_carrier	1592	2.413	0.5432	0.8321	6.998	1.622	0.1049
21	rs2838003	42649357	C	ADDxapoe_carrier	1555	1.533	0.267	0.9083	2.587	1.6	0.1097
21	rs67525224	42627220	C	ADDxapoe_carrier	1565	1.96	0.4218	0.8574	4.479	1.595	0.1107
21	rs67757006	42525663	A	ADDxapoe_carrier	1537	1.694	0.3352	0.8783	3.268	1.573	0.1157
21	rs57911156	42522469	C	ADDxapoe_carrier	1531	1.693	0.3351	0.8778	3.264	1.571	0.1162
21	rs2838002	42648767	C	ADDxapoe_carrier	1557	1.517	0.266	0.9005	2.554	1.566	0.1174
21	rs55853145	42524465	A	ADDxapoe_carrier	1535	1.69	0.3352	0.876	3.26	1.565	0.1176
21	rs62217917	42527354	A	ADDxapoe_carrier	1528	1.669	0.3357	0.8642	3.222	1.525	0.1272
21	rs11702001	42627969	A	ADDxapoe_carrier	1569	1.893	0.4225	0.8272	4.333	1.511	0.1308
21	rs1810868	42651481	A	ADDxapoe_carrier	1567	0.7696	0.1743	0.5469	1.083	-1.502	0.133
21	rs2012050	42651562	T	ADDxapoe_carrier	1531	1.372	0.2107	0.9078	2.073	1.501	0.1335
21	rs11700578	42522350	T	ADDxapoe_carrier	1527	1.324	0.1894	0.9131	1.919	1.48	0.1388
21	rs2183588	42626882	A	ADDxapoe_carrier	1583	1.393	0.2241	0.8979	2.162	1.479	0.1391
21	rs8134992	42624622	T	ADDxapoe_carrier	1561	2.233	0.5472	0.7641	6.526	1.468	0.1421
21	rs11701114	42522656	G	ADDxapoe_carrier	1531	1.32	0.1892	0.9107	1.912	1.466	0.1427
21	rs11700757	42522447	A	ADDxapoe_carrier	1529	1.316	0.1893	0.9082	1.908	1.451	0.1467
21	rs8133778	42642038	A	ADDxapoe_carrier	1558	1.445	0.2552	0.8765	2.384	1.444	0.1488
21	rs11700599	42522499	G	ADDxapoe_carrier	1530	1.312	0.1893	0.9054	1.902	1.435	0.1513
21	rs2837999	42630531	A	ADDxapoe_carrier	1547	1.417	0.2432	0.88	2.282	1.434	0.1516
21	rs2837989	42620119	A	ADDxapoe_carrier	1567	1.762	0.4206	0.7724	4.017	1.346	0.1783
21	rs9975388	42626362	C	ADDxapoe_carrier	1581	1.331	0.2178	0.8687	2.04	1.314	0.1889
21	rs2837985	42617756	G	ADDxapoe_carrier	1534	2.153	0.6255	0.6317	7.335	1.226	0.2203
21	rs8127120	42619274	G	ADDxapoe_carrier	1557	1.839	0.5074	0.6803	4.972	1.201	0.2298
21	rs2410415	42641402	C	ADDxapoe_carrier	1556	0.726	0.3023	0.4014	1.313	-1.059	0.2895
21	rs7278856	42598887	G	ADDxapoe_carrier	1545	1.377	0.3023	0.7616	2.491	1.059	0.2896
21	rs8134468	42638685	A	ADDxapoe_carrier	1532	0.7283	0.3077	0.3985	1.331	-1.03	0.3028
21	rs58867243	42644663	A	ADDxapoe_carrier	1568	1.667	0.5091	0.6148	4.523	1.004	0.3152
21	rs960230	42622479	A	ADDxapoe_carrier	1544	1.358	0.3257	0.7169	2.57	0.9384	0.348
21	rs4818228	42643772	A	ADDxapoe_carrier	1566	1.401	0.3609	0.6906	2.842	0.9341	0.3503
21	rs9808711	42642642	A	ADDxapoe_carrier	1589	1.393	0.3607	0.6869	2.825	0.9188	0.3582

21	rs4818229	42643968	T	ADDxapoe_carrier	1589	1.393	0.3607	0.6869	2.825	0.9188	0.3582
21	rs2838001	42646992	C	ADDxapoe_carrier	1580	1.368	0.3608	0.6743	2.774	0.8678	0.3855
21	rs2838000	42646518	A	ADDxapoe_carrier	1586	1.352	0.359	0.669	2.733	0.8404	0.4007
21	rs2837971	42601214	A	ADDxapoe_carrier	1561	1.291	0.305	0.7101	2.347	0.8374	0.4024
21	rs6517653	42534253	A	ADDxapoe_carrier	1585	1.578	0.5679	0.5185	4.804	0.8036	0.4216
21	rs11088544	42517484	C	ADDxapoe_carrier	1530	1.207	0.2474	0.7433	1.96	0.7604	0.447
21	rs2837990	42620149	A	ADDxapoe_carrier	1587	1.31	0.36	0.6472	2.654	0.7511	0.4526
21	rs11702409	42537012	G	ADDxapoe_carrier	1548	1.536	0.5741	0.4986	4.733	0.7479	0.4545
21	rs2837991	42620160	A	ADDxapoe_carrier	1587	1.309	0.36	0.6464	2.651	0.7479	0.4545
21	rs62219579	42643610	C	ADDxapoe_carrier	1556	0.5382	0.8333	0.1051	2.756	-0.7435	0.4572
21	rs11700718	42644982	C	ADDxapoe_carrier	1555	0.5387	0.8332	0.1052	2.758	-0.7425	0.4578
21	rs59836194	42634841	T	ADDxapoe_carrier	1566	0.5392	0.8333	0.1053	2.761	-0.7413	0.4585
21	rs2016240	42530260	T	ADDxapoe_carrier	1568	1.523	0.5685	0.4997	4.64	0.7398	0.4594
21	rs68087522	42642096	G	ADDxapoe_carrier	1559	0.5428	0.8332	0.106	2.779	-0.7334	0.4633
21	rs914173	42517552	C	ADDxapoe_carrier	1584	1.499	0.5601	0.5	4.494	0.7227	0.4699
21	rs7281814	42518444	G	ADDxapoe_carrier	1583	1.496	0.5602	0.4989	4.483	0.7186	0.4724
21	rs2837962	42518650	G	ADDxapoe_carrier	1582	1.452	0.5609	0.4838	4.36	0.6654	0.5058
21	rs2776343	42521267	A	ADDxapoe_carrier	1563	1.181	0.2496	0.7239	1.925	0.6651	0.506
21	rs914175	42517750	A	ADDxapoe_carrier	1588	1.444	0.5585	0.4831	4.314	0.6575	0.5109
21	rs11700778	42517399	A	ADDxapoe_carrier	1589	1.439	0.5585	0.4817	4.301	0.6523	0.5142
21	rs2898440	42517480	A	ADDxapoe_carrier	1589	1.439	0.5585	0.4816	4.3	0.6516	0.5147
21	rs6517659	42641982	T	ADDxapoe_carrier	1559	0.7836	0.3896	0.3651	1.682	-0.6258	0.5315
21	rs11088546	42532977	A	ADDxapoe_carrier	1585	0.9001	0.1776	0.6354	1.275	-0.5927	0.5534
21	rs11909506	42678742	G	ADDxapoe_carrier	1583	1.146	0.2301	0.7301	1.799	0.5925	0.5535
21	rs2776342	42521213	C	ADDxapoe_carrier	1567	1.152	0.2483	0.7083	1.875	0.5712	0.5679
21	rs13048452	42532909	G	ADDxapoe_carrier	1586	0.904	0.1776	0.6383	1.28	-0.5686	0.5697
21	rs35939063	42584621	G	ADDxapoe_carrier	1539	0.7938	0.4069	0.3576	1.762	-0.5677	0.5702
21	rs11700917	42517222	T	ADDxapoe_carrier	1531	1.369	0.5716	0.4464	4.196	0.5488	0.5831
21	rs7279020	42534031	T	ADDxapoe_carrier	1573	0.9107	0.1777	0.6429	1.29	-0.5265	0.5985
21	rs12627075	42520443	C	ADDxapoe_carrier	1541	0.9167	0.1702	0.6566	1.28	-0.5107	0.6095
21	rs2705572	42521161	G	ADDxapoe_carrier	1570	1.134	0.2478	0.6978	1.844	0.5084	0.6112
21	rs4816714	42574691	G	ADDxapoe_carrier	1537	0.7287	0.6371	0.2091	2.54	-0.4968	0.6194
21	rs4818222	42574696	G	ADDxapoe_carrier	1537	0.7287	0.6371	0.2091	2.54	-0.4968	0.6194
21	rs2150425	42531759	A	ADDxapoe_carrier	1577	0.9125	0.19	0.6288	1.324	-0.4817	0.63
21	rs9974272	42521711	G	ADDxapoe_carrier	1541	1.156	0.3066	0.6338	2.108	0.4727	0.6364
21	rs914174	42517678	A	ADDxapoe_carrier	1580	1.097	0.2037	0.7363	1.636	0.4566	0.6479
21	rs8127156	42543403	G	ADDxapoe_carrier	1593	1.108	0.2402	0.6919	1.774	0.4265	0.6698
21	rs9983938	42516357	G	ADDxapoe_carrier	1571	1.087	0.2043	0.7287	1.623	0.4106	0.6814
21	rs1001453	42515277	A	ADDxapoe_carrier	1532	1.116	0.2698	0.6576	1.893	0.4062	0.6846
21	rs1571732	42517559	G	ADDxapoe_carrier	1576	1.105	0.2481	0.6795	1.797	0.4028	0.6871
21	rs914176	42528192	G	ADDxapoe_carrier	1538	0.933	0.18	0.6556	1.328	-0.3855	0.6999
21	rs13052926	42585088	G	ADDxapoe_carrier	1555	0.8501	0.4326	0.3641	1.985	-0.3755	0.7073
21	rs876817	42528499	C	ADDxapoe_carrier	1569	1.087	0.2303	0.6918	1.706	0.3603	0.7186

21	rs3787933	42573589	G	ADDxapoe_carrier	1572	1.236	0.5896	0.389	3.924	0.3587	0.7198
21	rs6517654	42534328	C	ADDxapoe_carrier	1556	0.9113	0.2721	0.5346	1.553	-0.3412	0.7329
21	rs6517655	42536246	C	ADDxapoe_carrier	1572	1.081	0.23	0.6887	1.696	0.3384	0.7351
21	rs9982181	42599843	T	ADDxapoe_carrier	1556	0.8447	0.5035	0.3149	2.266	-0.3353	0.7374
21	rs62219580	42649211	G	ADDxapoe_carrier	1537	0.7456	0.9059	0.1263	4.402	-0.324	0.7459
21	rs35335714	42517341	C	ADDxapoe_carrier	1575	1.095	0.2824	0.6295	1.904	0.3212	0.748
21	rs9975636	42531333	A	ADDxapoe_carrier	1593	0.918	0.2719	0.5388	1.564	-0.3146	0.753
21	rs28654619	42536126	C	ADDxapoe_carrier	1586	0.9203	0.2719	0.5401	1.568	-0.3055	0.76
21	rs6517657	42584141	T	ADDxapoe_carrier	1521	1.15	0.4827	0.4464	2.962	0.2893	0.7724
21	rs73224118	42653246	T	ADDxapoe_carrier	1546	0.7734	0.9045	0.1314	4.554	-0.2841	0.7764
21	rs9976965	42679090	C	ADDxapoe_carrier	1590	0.9235	0.2815	0.5318	1.604	-0.2827	0.7774
21	rs12483323	42643845	T	ADDxapoe_carrier	1553	1.1	0.3589	0.5442	2.222	0.2644	0.7915
21	rs7279273	42664100	C	ADDxapoe_carrier	1579	0.9574	0.166	0.6915	1.326	-0.2623	0.7931
21	rs2837964	42520646	A	ADDxapoe_carrier	1547	1.067	0.2492	0.6548	1.739	0.2605	0.7945
21	rs8134160	42541202	T	ADDxapoe_carrier	1527	1.063	0.2415	0.6622	1.706	0.2529	0.8003
21	rs62217923	42539293	G	ADDxapoe_carrier	1525	1.063	0.2433	0.6598	1.712	0.251	0.8019
21	rs2297271	42520327	G	ADDxapoe_carrier	1564	0.9586	0.1687	0.6887	1.334	-0.2505	0.8022
21	rs2248555	42520337	G	ADDxapoe_carrier	1564	0.9586	0.1687	0.6887	1.334	-0.2505	0.8022
21	rs1041445	42515737	T	ADDxapoe_carrier	1526	0.9415	0.2534	0.573	1.547	-0.2377	0.8121
21	rs9980363	42520134	C	ADDxapoe_carrier	1570	0.9373	0.2746	0.5472	1.606	-0.2356	0.8137
21	rs10775668	42678029	T	ADDxapoe_carrier	1589	0.9385	0.2792	0.543	1.622	-0.2272	0.8202
21	rs1571733	42518034	A	ADDxapoe_carrier	1550	0.9007	0.4607	0.3651	2.222	-0.2271	0.8204
21	rs9976216	42679037	G	ADDxapoe_carrier	1596	0.9407	0.2792	0.5442	1.626	-0.2192	0.8265
21	rs9976125	42678988	G	ADDxapoe_carrier	1595	0.9408	0.2792	0.5443	1.626	-0.2187	0.8269
21	rs9976433	42679010	A	ADDxapoe_carrier	1595	0.9408	0.2792	0.5443	1.626	-0.2187	0.8269
21	rs9976217	42679042	G	ADDxapoe_carrier	1595	0.9408	0.2792	0.5443	1.626	-0.2186	0.827
21	rs4816716	42674021	A	ADDxapoe_carrier	1524	0.88	0.5983	0.2724	2.843	-0.2137	0.8308
21	rs67438118	42669465	A	ADDxapoe_carrier	1524	0.882	0.5984	0.273	2.85	-0.2098	0.8338
21	rs2210277	42536308	C	ADDxapoe_carrier	1588	1.048	0.2247	0.6747	1.628	0.2088	0.8346
21	rs2837961	42518599	C	ADDxapoe_carrier	1581	0.9701	0.169	0.6965	1.351	-0.1796	0.8575
21	rs28360666	42517239	T	ADDxapoe_carrier	1581	0.9707	0.1693	0.6966	1.353	-0.1756	0.8606
21	rs726980	42635676	A	ADDxapoe_carrier	1559	1.108	0.5983	0.3429	3.579	0.1713	0.864
21	rs11701489	42517643	A	ADDxapoe_carrier	1586	1.047	0.2707	0.6161	1.78	0.1704	0.8647
21	rs67837204	42632585	T	ADDxapoe_carrier	1565	0.8602	0.8977	0.1481	4.997	-0.1678	0.8668
21	rs62217915	42519037	C	ADDxapoe_carrier	1591	0.9284	0.4533	0.3818	2.257	-0.1638	0.8699
21	rs58404061	42638931	A	ADDxapoe_carrier	1567	0.8652	0.8976	0.149	5.025	-0.1613	0.8719
21	rs9980188	42664366	G	ADDxapoe_carrier	1587	0.9744	0.1657	0.7042	1.348	-0.1566	0.8755
21	rs13049769	42664689	C	ADDxapoe_carrier	1587	0.9744	0.1657	0.7042	1.348	-0.1566	0.8755
21	rs34019132	42583154	T	ADDxapoe_carrier	1593	1.067	0.4176	0.4707	2.419	0.1554	0.8765
21	rs9984070	42516327	T	ADDxapoe_carrier	1577	1.041	0.2707	0.6126	1.77	0.1494	0.8812
21	rs28360503	42583804	C	ADDxapoe_carrier	1581	1.063	0.4179	0.4689	2.412	0.1473	0.8829
21	rs11702600	42524832	G	ADDxapoe_carrier	1575	0.9664	0.2862	0.5515	1.693	-0.1195	0.9049
21	rs6517656	42583738	A	ADDxapoe_carrier	1529	1.046	0.4342	0.4466	2.45	0.1034	0.9177

21	rs13049454	42526520	A	ADDxapoe_carrier	1580	0.977	0.286	0.5577	1.711	-0.08149	0.935
21	rs7276900	42532075	A	ADDxapoe_carrier	1569	1.018	0.2243	0.6558	1.58	0.07927	0.9368
21	rs11559045	42652831	T	ADDxapoe_carrier	1549	0.9513	0.7398	0.2232	4.055	-0.06753	0.9462
21	rs68183718	42678791	T	ADDxapoe_carrier	1585	0.9647	0.5875	0.305	3.051	-0.06118	0.9512
21	rs880370	42527504	A	ADDxapoe_carrier	1541	0.9939	0.171	0.7109	1.389	-0.036	0.9713
21	rs11701197	42522788	A	ADDxapoe_carrier	1576	0.9901	0.2856	0.5657	1.733	-0.03474	0.9723
21	rs2007397	42516192	A	ADDxapoe_carrier	1559	0.9933	0.1972	0.6748	1.462	-0.03421	0.9727
21	rs9305726	42525272	A	ADDxapoe_carrier	1592	0.9916	0.2855	0.5667	1.735	-0.02957	0.9764
21	rs9305727	42525411	G	ADDxapoe_carrier	1592	0.9916	0.2855	0.5667	1.735	-0.02957	0.9764
21	rs9305728	42525448	A	ADDxapoe_carrier	1592	0.9916	0.2855	0.5667	1.735	-0.02957	0.9764
21	rs9982271	42519389	T	ADDxapoe_carrier	1563	0.992	0.2726	0.5814	1.693	-0.02934	0.9766
21	rs11701356	42522963	C	ADDxapoe_carrier	1597	0.9935	0.2855	0.5678	1.738	-0.02282	0.9818
21	rs10154102	42523427	G	ADDxapoe_carrier	1597	0.9935	0.2855	0.5678	1.738	-0.02286	0.9818
21	rs73905316	42668059	T	ADDxapoe_carrier	1527	1736000	647.6	0	inf	0.02218	0.9823
21	rs11702752	42524741	C	ADDxapoe_carrier	1593	0.9938	0.2855	0.5679	1.739	-0.02175	0.9826
21	rs11702604	42524870	T	ADDxapoe_carrier	1593	0.9938	0.2855	0.5679	1.739	-0.02175	0.9826
21	rs10154234	42525007	A	ADDxapoe_carrier	1593	0.9938	0.2855	0.5679	1.739	-0.02175	0.9826
21	rs9305724	42525185	G	ADDxapoe_carrier	1593	0.9938	0.2855	0.5679	1.739	-0.02175	0.9826
21	rs9305725	42525257	C	ADDxapoe_carrier	1592	0.9938	0.2855	0.5679	1.739	-0.02179	0.9826
21	rs7276719	42670165	A	ADDxapoe_carrier	1526	35550000	836.1	0	inf	0.02079	0.9834
21	rs11702590	42524628	A	ADDxapoe_carrier	1592	0.9951	0.2855	0.5686	1.741	-0.01737	0.9861
21	rs11702163	42524513	C	ADDxapoe_carrier	1591	0.9951	0.2855	0.5687	1.741	-0.01721	0.9863
21	rs9305731	42598396	G	ADDxapoe_carrier	1559	1.007	0.4558	0.4122	2.461	0.0155	0.9876
21	rs73905312	42663603	A	ADDxapoe_carrier	1528	4190000	1024	0	inf	0.01489	0.9881
21	rs10154104	42523511	G	ADDxapoe_carrier	1595	0.9963	0.2855	0.5694	1.743	-0.01299	0.9896
21	rs11701851	42523852	T	ADDxapoe_carrier	1592	0.9974	0.2855	0.57	1.745	-0.00901	0.9928
21	rs7277920	42575415	C	ADDxapoe_carrier	1568	0.9958	0.6072	0.3029	3.274	-0.0069	0.9945

Supplementary Table 4

CHR	SNP	BP	A1	TEST	NMISS	BETA	SE	L95	U95	STAT	P
21	rs2837994	42624124	A	ADDxapoe_carrier	48	-139.2	33.83	-205.5	-72.86	-4.114	0.000183
21	rs11559045	42652831	T	ADDxapoe_carrier	48	-133.9	33.09	-198.8	-69.08	-4.048	0.000224
21	rs1077117	42599364	G	ADDxapoe_carrier	47	-65.59	21.27	-107.3	-23.9	-3.084	0.003696
21	rs2837971	42601214	A	ADDxapoe_carrier	47	-65.59	21.27	-107.3	-23.9	-3.084	0.003696
21	rs6517661	42653649	C	ADDxapoe_carrier	48	-68.01	22.23	-111.6	-24.44	-3.059	0.003901
21	rs7279020	42534031	T	ADDxapoe_carrier	47	57.95	23.73	11.44	104.5	2.442	0.01913
21	rs77395637	42627316	A	ADDxapoe_carrier	47	88.78	37.47	15.34	162.2	2.369	0.02274
21	rs67525224	42627220	C	ADDxapoe_carrier	48	-52.06	23.14	-97.41	-6.715	-2.25	0.02987
21	rs28629220	42597387	C	ADDxapoe_carrier	47	88.81	39.61	11.18	166.4	2.242	0.03056
21	rs2837989	42620119	A	ADDxapoe_carrier	47	-49.2	23.31	-94.89	-3.502	-2.11	0.04114
21	rs2837991	42620160	A	ADDxapoe_carrier	47	-49.42	23.69	-95.85	-2.991	-2.086	0.04338
21	rs7281337	42655459	G	ADDxapoe_carrier	47	-45.67	22.93	-90.61	-0.7368	-1.992	0.05322
21	rs12482462	42626851	T	ADDxapoe_carrier	47	28.71	15.16	-1.012	58.43	1.893	0.06558
21	rs2837975	42608854	A	ADDxapoe_carrier	48	-29.84	17.16	-63.47	3.779	-1.74	0.08942
21	rs77240271	42642405	A	ADDxapoe_carrier	48	74.28	43.65	-11.28	159.8	1.702	0.09642
21	rs2183588	42626882	A	ADDxapoe_carrier	48	-24.68	15.18	-54.43	5.072	-1.626	0.1116
21	rs73222287	42630024	G	ADDxapoe_carrier	47	75.51	46.76	-16.14	167.2	1.615	0.1142
21	rs75748434	42630703	T	ADDxapoe_carrier	48	70.3	44.31	-16.55	157.1	1.586	0.1203
21	rs4818227	42643729	A	ADDxapoe_carrier	48	-21.69	14.98	-51.05	7.673	-1.448	0.1553
21	rs2838002	42648767	C	ADDxapoe_carrier	48	-22.16	15.42	-52.38	8.069	-1.437	0.1584
21	rs7279273	42664100	C	ADDxapoe_carrier	48	25.67	18.38	-10.35	61.68	1.397	0.17
21	rs10154086	42659692	T	ADDxapoe_carrier	47	23.26	16.89	-9.852	56.36	1.377	0.1762
21	rs9981553	42620680	T	ADDxapoe_carrier	46	22.43	16.35	-9.613	54.47	1.372	0.1779
21	rs2837993	42620714	T	ADDxapoe_carrier	46	22.43	16.35	-9.613	54.47	1.372	0.1779
21	rs8133778	42642038	A	ADDxapoe_carrier	48	-19.99	15.69	-50.73	10.76	-1.274	0.2098
21	rs9980188	42664366	G	ADDxapoe_carrier	48	20.7	17.54	-13.67	55.07	1.18	0.2447
21	rs34996007	42644885	T	ADDxapoe_carrier	48	43.79	38.47	-31.6	119.2	1.138	0.2616
21	rs2012050	42651562	T	ADDxapoe_carrier	46	-15.18	13.97	-42.55	12.2	-1.087	0.2839
21	rs9975636	42531333	A	ADDxapoe_carrier	48	-22.28	21.68	-64.77	20.22	-1.028	0.3102
21	rs9975323	42666463	C	ADDxapoe_carrier	48	19.47	19.02	-17.82	56.75	1.023	0.3121
21	rs9305734	42661314	A	ADDxapoe_carrier	46	20.02	19.73	-18.65	58.69	1.015	0.3164
21	rs9305735	42661365	G	ADDxapoe_carrier	46	20.02	19.73	-18.65	58.69	1.015	0.3164
21	rs9305724	42525185	G	ADDxapoe_carrier	48	-21.55	22.3	-65.26	22.15	-0.9665	0.3395
21	rs11702001	42627969	A	ADDxapoe_carrier	48	-21.13	24.95	-70.03	27.77	-0.8469	0.402
21	rs79171038	42654472	G	ADDxapoe_carrier	48	33.14	40.07	-45.4	111.7	0.827	0.413
21	rs1999331	42654497	A	ADDxapoe_carrier	47	23.75	29.21	-33.5	80.99	0.813	0.421
21	rs2837965	42567637	T	ADDxapoe_carrier	48	-12.45	16.06	-43.93	19.03	-0.7751	0.4427

21	rs6517654	42534328	C	ADDxapoe_carrier	48	14.64	19.14	-22.88	52.15	0.7646	0.4489
21	rs28654619	42536126	C	ADDxapoe_carrier	48	14.64	19.14	-22.88	52.15	0.7646	0.4489
21	rs34019132	42583154	T	ADDxapoe_carrier	48	-13.62	18.97	-50.79	23.56	-0.7178	0.4769
21	rs28360503	42583804	C	ADDxapoe_carrier	48	-13.62	18.97	-50.79	23.56	-0.7178	0.4769
21	rs6517657	42584141	T	ADDxapoe_carrier	48	-13.62	18.97	-50.79	23.56	-0.7178	0.4769
21	rs2837996	42626706	T	ADDxapoe_carrier	48	-10.82	16.02	-42.21	20.57	-0.6756	0.5031
21	rs9978431	42645737	T	ADDxapoe_carrier	47	-11.42	17.76	-46.22	23.39	-0.6429	0.524
21	rs9305725	42525257	C	ADDxapoe_carrier	48	12.12	19.22	-25.55	49.79	0.6305	0.5319
21	rs2410406	42581529	C	ADDxapoe_carrier	48	-10.77	17.69	-45.45	23.9	-0.6089	0.5459
21	rs1077339	42581575	G	ADDxapoe_carrier	48	-10.77	17.69	-45.45	23.9	-0.6089	0.5459
21	rs1077338	42581594	A	ADDxapoe_carrier	48	-10.77	17.69	-45.45	23.9	-0.6089	0.5459
21	rs737288	42581637	T	ADDxapoe_carrier	48	-10.77	17.69	-45.45	23.9	-0.6089	0.5459
21	rs737287	42581703	T	ADDxapoe_carrier	48	-10.77	17.69	-45.45	23.9	-0.6089	0.5459
21	rs8134468	42638685	A	ADDxapoe_carrier	48	-13.41	22.07	-56.66	29.85	-0.6075	0.5469
21	rs2297271	42520327	G	ADDxapoe_carrier	48	-13.48	22.88	-58.33	31.37	-0.5891	0.559
21	rs35291683	42576275	A	ADDxapoe_carrier	47	-19.64	33.89	-86.06	46.78	-0.5795	0.5655
21	rs35939063	42584621	G	ADDxapoe_carrier	46	-10.53	19.08	-47.92	26.86	-0.552	0.5841
21	rs914182	42582981	G	ADDxapoe_carrier	47	-9.84	18.73	-46.56	26.88	-0.5253	0.6023
21	rs66871207	42660588	G	ADDxapoe_carrier	48	-12.51	26.9	-65.23	40.22	-0.4649	0.6444
21	rs9984070	42516327	T	ADDxapoe_carrier	46	7.761	18.48	-28.46	43.98	0.42	0.6768
21	rs9982271	42519389	T	ADDxapoe_carrier	46	7.761	18.48	-28.46	43.98	0.42	0.6768
21	rs7277920	42575415	C	ADDxapoe_carrier	48	7.515	20.49	-32.65	47.68	0.3667	0.7157
21	rs12149	42647821	T	ADDxapoe_carrier	48	4.941	15.08	-24.61	34.49	0.3277	0.7448
21	rs12482242	42648821	G	ADDxapoe_carrier	48	4.941	15.08	-24.61	34.49	0.3277	0.7448
21	rs117504953	42623780	C	ADDxapoe_carrier	48	18.33	57.79	-94.93	131.6	0.3172	0.7527
21	rs9976426	42641913	T	ADDxapoe_carrier	48	4.675	15.26	-25.23	34.58	0.3065	0.7608
21	rs9305733	42642699	C	ADDxapoe_carrier	48	4.675	15.26	-25.23	34.58	0.3065	0.7608
21	rs62219580	42649211	G	ADDxapoe_carrier	48	8.802	29.24	-48.51	66.11	0.301	0.7649
21	rs1810868	42651481	A	ADDxapoe_carrier	46	4.899	16.94	-28.3	38.1	0.2893	0.7739
21	rs68087522	42642096	G	ADDxapoe_carrier	48	8.036	29.58	-49.94	66.01	0.2717	0.7872
21	rs62219579	42643610	C	ADDxapoe_carrier	48	8.036	29.58	-49.94	66.01	0.2717	0.7872
21	rs9808711	42642642	A	ADDxapoe_carrier	48	-3.13	17.56	-37.54	31.28	-0.1783	0.8594
21	rs914186	42619657	G	ADDxapoe_carrier	46	2.36	20.75	-38.3	43.02	0.1137	0.91
21	rs2838004	42650654	G	ADDxapoe_carrier	47	0.8652	17.63	-33.69	35.42	0.04907	0.9611
21	rs67438118	42669465	A	ADDxapoe_carrier	48	-0.9476	25.35	-50.63	48.74	-0.03738	0.9704
21	rs2837999	42630531	A	ADDxapoe_carrier	48	0.4381	18.68	-36.18	37.06	0.02345	0.9814
21	rs67484863	42664418	A	ADDxapoe_carrier	48	0.596	25.35	-49.09	50.29	0.02351	0.9814
21	rs4818229	42643968	T	ADDxapoe_carrier	48	-0.4269	18.8	-37.28	36.43	-0.0227	0.982

Supplementary Table 5

CHR	SNP	BP	A1	TEST	NMISS	BETA	SE	L95	U95	STAT	P
21	rs9980146	42659015	T	ADDxapoe_carrier	103	35.56	15.12	5.936	65.19	2.353	0.02068
21	rs1072869	42657548	C	ADDxapoe_carrier	106	32.69	15.1	3.093	62.28	2.165	0.03281
21	rs67837204	42632585	T	ADDxapoe_carrier	106	-51.76	24.94	-100.6	-2.87	-2.075	0.04058
21	rs67237909	42616201	A	ADDxapoe_carrier	102	-45.19	22.21	-88.71	-1.664	-2.035	0.04465
21	rs4818229	42643968	T	ADDxapoe_carrier	102	-37.7	18.61	-74.17	-1.231	-2.026	0.04555
21	rs9978431	42645737	T	ADDxapoe_carrier	104	-27.24	14.26	-55.18	0.7022	-1.911	0.059
21	rs12627066	42618664	T	ADDxapoe_carrier	105	-46.8	30	-105.6	12	-1.56	0.122
21	rs9974272	42521711	G	ADDxapoe_carrier	106	24.49	17.27	-9.357	58.33	1.418	0.1593
21	rs3827211	42619254	G	ADDxapoe_carrier	105	17.82	14.18	-9.978	45.62	1.256	0.212
21	rs2837981	42616005	C	ADDxapoe_carrier	105	-20.02	16.22	-51.81	11.78	-1.234	0.2202
21	rs960230	42622479	A	ADDxapoe_carrier	106	-21.65	17.56	-56.06	12.77	-1.233	0.2206
21	rs2297271	42520327	G	ADDxapoe_carrier	105	-18.63	15.17	-48.36	11.1	-1.228	0.2223
21	rs9984070	42516327	T	ADDxapoe_carrier	103	20.92	17.46	-13.31	55.15	1.198	0.234
21	rs35335714	42517341	C	ADDxapoe_carrier	103	20.92	17.46	-13.31	55.15	1.198	0.234
21	rs9983496	42586130	C	ADDxapoe_carrier	106	-17.69	14.98	-47.05	11.68	-1.181	0.2406
21	rs9305725	42525257	C	ADDxapoe_carrier	104	21.53	18.29	-14.32	57.37	1.177	0.2421
21	rs11701197	42522788	A	ADDxapoe_carrier	106	19.17	17.47	-15.07	53.41	1.097	0.2752
21	rs11701356	42522963	C	ADDxapoe_carrier	106	19.17	17.47	-15.07	53.41	1.097	0.2752
21	rs13048452	42532909	G	ADDxapoe_carrier	103	15.37	14.17	-12.4	43.15	1.085	0.2808
21	rs9982271	42519389	T	ADDxapoe_carrier	104	18.88	17.62	-15.65	53.41	1.071	0.2866
21	rs9975636	42531333	A	ADDxapoe_carrier	106	18.97	17.94	-16.19	54.14	1.057	0.2929
21	rs2006737	42657187	G	ADDxapoe_carrier	106	-15.09	14.7	-43.91	13.73	-1.026	0.3074
21	rs2183588	42626882	A	ADDxapoe_carrier	105	-14.3	14.04	-41.82	13.23	-1.018	0.3111
21	rs7279020	42534031	T	ADDxapoe_carrier	105	14.6	14.58	-13.97	43.17	1.002	0.319
21	rs11701489	42517643	A	ADDxapoe_carrier	103	17.27	17.38	-16.79	51.33	0.9938	0.3228
21	rs28654619	42536126	C	ADDxapoe_carrier	105	18.11	18.89	-18.91	55.13	0.9588	0.34
21	rs7277920	42575415	C	ADDxapoe_carrier	104	-20.7	22.48	-64.75	23.35	-0.9209	0.3594
21	rs67484863	42664418	A	ADDxapoe_carrier	102	-22.61	24.98	-71.57	26.34	-0.9054	0.3675
21	rs914186	42619657	G	ADDxapoe_carrier	105	-12.85	14.75	-41.75	16.06	-0.8709	0.3859
21	rs7281733	42655515	A	ADDxapoe_carrier	103	-17.43	20.21	-57.05	22.18	-0.8624	0.3906
21	rs11909506	42678742	G	ADDxapoe_carrier	104	-13.28	16.28	-45.19	18.63	-0.8158	0.4166
21	rs6517654	42534328	C	ADDxapoe_carrier	104	15.49	19.3	-22.33	53.31	0.8029	0.424
21	rs67438118	42669465	A	ADDxapoe_carrier	103	-20.07	25.14	-69.35	29.21	-0.7981	0.4268
21	rs4816716	42674021	A	ADDxapoe_carrier	103	-20.07	25.14	-69.35	29.21	-0.7981	0.4268
21	rs914187	42619749	T	ADDxapoe_carrier	105	-11.01	14.67	-39.76	17.75	-0.7503	0.4549
21	rs62219580	42649211	G	ADDxapoe_carrier	106	20.31	27.86	-34.3	74.92	0.729	0.4677
21	rs74477222	42636501	T	ADDxapoe_carrier	105	-29.39	41.08	-109.9	51.12	-0.7155	0.476

21	rs960231	42622657	G	ADDxapoe_carrier	106	-9.593	13.99	-37.02	17.83	-0.6856	0.4946
21	rs73905312	42663603	A	ADDxapoe_carrier	103	20.21	29.92	-38.43	78.85	0.6755	0.501
21	rs77270082	42531725	T	ADDxapoe_carrier	105	22.01	33.49	-43.62	87.65	0.6573	0.5125
21	rs9976125	42678988	G	ADDxapoe_carrier	106	-11.78	18.79	-48.61	25.06	-0.6266	0.5324
21	rs10775668	42678029	T	ADDxapoe_carrier	105	-11.73	19	-48.98	25.51	-0.6175	0.5383
21	rs7281337	42655459	G	ADDxapoe_carrier	105	-11.94	19.5	-50.16	26.28	-0.6122	0.5418
21	rs75748434	42630703	T	ADDxapoe_carrier	105	-24.55	41.04	-105	55.89	-0.5982	0.5511
21	rs2150425	42531759	A	ADDxapoe_carrier	106	8.743	15.16	-20.96	38.45	0.5768	0.5654
21	rs75896199	42678194	G	ADDxapoe_carrier	104	17.77	30.94	-42.87	78.41	0.5743	0.5671
21	rs57347504	42678481	G	ADDxapoe_carrier	104	17.77	30.94	-42.87	78.41	0.5743	0.5671
21	rs66871207	42660588	G	ADDxapoe_carrier	106	-14.35	25.19	-63.73	35.02	-0.5697	0.5702
21	rs7281814	42518444	G	ADDxapoe_carrier	104	-9.861	19.11	-47.32	27.6	-0.5159	0.6071
21	rs2837962	42518650	G	ADDxapoe_carrier	104	-9.861	19.11	-47.32	27.6	-0.5159	0.6071
21	rs11701756	42632770	A	ADDxapoe_carrier	104	15.6	31.16	-45.47	76.67	0.5008	0.6177
21	rs76228446	42632720	T	ADDxapoe_carrier	105	-17.34	40.55	-96.8	62.13	-0.4276	0.6699
21	rs62217922	42532753	A	ADDxapoe_carrier	105	13.88	34.43	-53.6	81.36	0.4032	0.6877
21	rs68183718	42678791	T	ADDxapoe_carrier	105	-9.18	23.82	-55.86	37.5	-0.3854	0.7007
21	rs28360503	42583804	C	ADDxapoe_carrier	106	7.942	22.63	-36.41	52.3	0.3509	0.7264
21	rs6517657	42584141	T	ADDxapoe_carrier	106	7.942	22.63	-36.41	52.3	0.3509	0.7264
21	rs34996007	42644885	T	ADDxapoe_carrier	104	10.06	31.55	-51.78	71.9	0.3187	0.7506
21	rs34019132	42583154	T	ADDxapoe_carrier	105	5.623	22.51	-38.49	49.73	0.2498	0.8032
21	rs56354888	42568152	A	ADDxapoe_carrier	104	4.791	19.74	-33.9	43.48	0.2427	0.8088
21	rs75000682	42662479	A	ADDxapoe_carrier	102	6.338	29.89	-52.24	64.91	0.2121	0.8325
21	rs2837975	42608854	A	ADDxapoe_carrier	106	-2.86	15.66	-33.55	27.83	-0.1826	0.8555
21	rs11702409	42537012	G	ADDxapoe_carrier	105	-3.461	19.09	-40.87	33.95	-0.1813	0.8565
21	rs6517661	42653649	C	ADDxapoe_carrier	105	-3.197	31.27	-64.49	58.09	-0.1022	0.9188
21	rs1571732	42517559	G	ADDxapoe_carrier	105	-0.3017	17.07	-33.76	33.15	-0.01767	0.9859
21	rs914181	42582752	G	ADDxapoe_carrier	105	-0.1724	16.1	-31.73	31.38	-0.01071	0.9915

Supplementary Table 6

CHR	SNP	BP	A1	TEST	NMISS	BETA	SE	L95	U95	STAT	P
21	rs9980146	42659015	T	ADDxapoe_carrier	56	31.7	23.01	-13.4	76.8	1.378	0.1745
21	rs2006741	42657221	C	ADDxapoe_carrier	55	-33.04	28.95	-89.77	23.7	-1.141	0.2594
21	rs13048452	42532909	G	ADDxapoe_carrier	55	22.27	20.29	-17.5	62.04	1.097	0.2779
21	rs11088546	42532977	A	ADDxapoe_carrier	56	21.59	20.47	-18.53	61.7	1.055	0.2967
21	rs7279020	42534031	T	ADDxapoe_carrier	56	21.59	20.47	-18.53	61.7	1.055	0.2967
21	rs12627075	42520443	C	ADDxapoe_carrier	54	-21.7	24.57	-69.85	26.46	-0.8831	0.3817
21	rs74977559	42613255	G	ADDxapoe_carrier	55	30.77	38.92	-45.5	107	0.7908	0.433
21	rs2837989	42620119	A	ADDxapoe_carrier	56	-21.47	28.4	-77.13	34.19	-0.7559	0.4533
21	rs6517659	42641982	T	ADDxapoe_carrier	56	-21.08	28.29	-76.52	34.35	-0.7454	0.4596
21	rs2248555	42520337	G	ADDxapoe_carrier	54	-15.5	21.56	-57.76	26.76	-0.7189	0.4757
21	rs73222273	42613545	G	ADDxapoe_carrier	55	23.55	35.07	-45.18	92.27	0.6715	0.5051
21	rs2297271	42520327	G	ADDxapoe_carrier	56	-11.96	21.44	-53.97	30.06	-0.5577	0.5796
21	rs2837961	42518599	C	ADDxapoe_carrier	55	-12.54	22.52	-56.68	31.59	-0.557	0.5801
21	rs8134468	42638685	A	ADDxapoe_carrier	56	-10.98	23.39	-56.83	34.86	-0.4696	0.6407
21	rs2837996	42626706	T	ADDxapoe_carrier	56	9.785	23.42	-36.11	55.68	0.4179	0.6779
21	rs75000682	42662479	A	ADDxapoe_carrier	56	24.56	60.93	-94.86	144	0.4031	0.6886
21	rs73905312	42663603	A	ADDxapoe_carrier	56	24.56	60.93	-94.86	144	0.4031	0.6886
21	rs73905316	42668059	T	ADDxapoe_carrier	56	24.56	60.93	-94.86	144	0.4031	0.6886
21	rs2183588	42626882	A	ADDxapoe_carrier	56	9.482	23.66	-36.89	55.85	0.4008	0.6903
21	rs9982271	42519389	T	ADDxapoe_carrier	54	9.231	24.55	-38.89	57.35	0.376	0.7086
21	rs62219580	42649211	G	ADDxapoe_carrier	56	-12.69	35.33	-81.95	56.56	-0.3593	0.7209
21	rs11701157	42651480	A	ADDxapoe_carrier	56	-12.69	35.33	-81.95	56.56	-0.3593	0.7209
21	rs3827211	42619254	G	ADDxapoe_carrier	55	9.756	30.28	-49.6	69.11	0.3222	0.7487
21	rs2837964	42520646	A	ADDxapoe_carrier	56	7.645	24.69	-40.75	56.04	0.3096	0.7581
21	rs2837994	42624124	A	ADDxapoe_carrier	54	10.36	33.86	-56.01	76.73	0.3059	0.761
21	rs3787933	42573589	G	ADDxapoe_carrier	56	8.352	27.39	-45.33	62.04	0.3049	0.7617
21	rs9305727	42525411	G	ADDxapoe_carrier	55	6.737	24.43	-41.15	54.63	0.2757	0.784
21	rs9305728	42525448	A	ADDxapoe_carrier	55	6.737	24.43	-41.15	54.63	0.2757	0.784
21	rs7277920	42575415	C	ADDxapoe_carrier	55	7.344	28.05	-47.64	62.33	0.2618	0.7946
21	rs9980363	42520134	C	ADDxapoe_carrier	56	4.825	24.2	-42.6	52.25	0.1994	0.8428
21	rs3787937	42609893	G	ADDxapoe_carrier	55	5.544	28.33	-49.98	61.07	0.1957	0.8457
21	rs74594237	42529023	A	ADDxapoe_carrier	54	7.409	41.33	-73.59	88.41	0.1793	0.8585
21	rs11702600	42524832	G	ADDxapoe_carrier	56	3.894	23.83	-42.81	50.6	0.1634	0.8709
21	rs11702604	42524870	T	ADDxapoe_carrier	56	3.894	23.83	-42.81	50.6	0.1634	0.8709
21	rs10154234	42525007	A	ADDxapoe_carrier	56	3.894	23.83	-42.81	50.6	0.1634	0.8709
21	rs9305726	42525272	A	ADDxapoe_carrier	56	3.894	23.83	-42.81	50.6	0.1634	0.8709
21	rs11701356	42522963	C	ADDxapoe_carrier	55	3.479	24.51	-44.57	51.53	0.1419	0.8877

21	rs2837993	42620714	T	ADDxapoe_carrier	54	-3.916	30.21	-63.12	55.29	-0.1296	0.8974
21	rs2837987	42618403	G	ADDxapoe_carrier	55	-3.745	31.37	-65.23	57.74	-0.1194	0.9055
21	rs73224110	42650666	A	ADDxapoe_carrier	56	5.329	52.84	-98.24	108.9	0.1009	0.9201
21	rs62217922	42532753	A	ADDxapoe_carrier	53	-2.004	40.74	-81.84	77.84	-0.04919	0.961
21	rs75306103	42640410	A	ADDxapoe_carrier	55	-2.877	65.94	-132.1	126.4	-0.04363	0.9654
21	rs74477222	42636501	T	ADDxapoe_carrier	56	-1.221	64.41	-127.5	125	-0.01896	0.985
21	rs58534150	42640903	T	ADDxapoe_carrier	56	-1.221	64.41	-127.5	125	-0.01896	0.985
21	rs11701197	42522788	A	ADDxapoe_carrier	56	0.08317	23.82	-46.6	46.76	0.003492	0.9972
21	rs10154102	42523427	G	ADDxapoe_carrier	56	0.08317	23.82	-46.6	46.76	0.003492	0.9972
21	rs10154104	42523511	G	ADDxapoe_carrier	56	0.08317	23.82	-46.6	46.76	0.003492	0.9972
21	rs11702590	42524628	A	ADDxapoe_carrier	56	0.08317	23.82	-46.6	46.76	0.003492	0.9972

Supplementary Table 7

SNP	BP	CHR	A1	BETA	SE	L95	U95	STAT	P	LOCATION	GENESYMBOL	CADD	AdjP
rs2012050	42651562	21	T	0.4174	0.2039	0.01771	0.8172	2.047	0.0425	intergenic	NA	4.184	1
rs11700578	42522350	21	C	-0.3	0.169	-0.6311	0.03118	-1.775	0.07788	intergenic	NA	1.044	1
rs11700757	42522447	21	T	-0.3	0.169	-0.6311	0.03118	-1.775	0.07788	intergenic	NA	0.072	1
rs11700599	42522499	21	C	-0.3	0.169	-0.6311	0.03118	-1.775	0.07788	intergenic	NA	8.106	1
rs11701114	42522656	21	A	-0.3	0.169	-0.6311	0.03118	-1.775	0.07788	intergenic	NA	0.019	1
rs6517654	42534328	21	C	0.3401	0.1968	-0.0456	0.7257	1.728	0.08602	intergenic	NA	0.441	1
rs28654619	42536126	21	C	0.3377	0.1962	-0.04689	0.7222	1.721	0.08729	intergenic	NA	3.37	1
rs9975636	42531333	21	A	0.3338	0.1956	-0.0495	0.7171	1.707	0.08988	intergenic	NA	1.541	1
rs11702409	42537012	21	G	0.571	0.3558	-0.1264	1.268	1.605	0.1107	intergenic	NA	0.788	1
rs2410415	42641402	21	C	-0.3414	0.2473	-0.8261	0.1433	-1.38	0.1696	intron	BACE2	5.85	1
rs13049454	42526520	21	A	0.2705	0.2006	-0.1227	0.6637	1.348	0.1795	intergenic	NA	2.152	1
rs10154104	42523511	21	G	0.2679	0.1999	-0.124	0.6598	1.34	0.1823	intergenic	NA	1.873	1
rs11701851	42523852	21	T	0.2676	0.2006	-0.1255	0.6608	1.334	0.1841	intergenic	NA	0.831	1
rs11702163	42524513	21	C	0.2676	0.2006	-0.1255	0.6608	1.334	0.1841	intergenic	NA	1.858	1
rs11702590	42524628	21	A	0.2676	0.2006	-0.1255	0.6608	1.334	0.1841	intergenic	NA	6.287	1
rs11702752	42524741	21	C	0.2676	0.2006	-0.1255	0.6608	1.334	0.1841	intergenic	NA	0.789	1
rs11702604	42524870	21	T	0.2676	0.2006	-0.1255	0.6608	1.334	0.1841	intergenic	NA	4.253	1
rs10154234	42525007	21	A	0.2676	0.2006	-0.1255	0.6608	1.334	0.1841	intergenic	NA	3.608	1
rs9305724	42525185	21	G	0.2676	0.2006	-0.1255	0.6608	1.334	0.1841	intergenic	NA	1.61	1
rs9305725	42525257	21	C	0.2676	0.2006	-0.1255	0.6608	1.334	0.1841	intergenic	NA	1.78	1
rs9305726	42525272	21	A	0.2676	0.2006	-0.1255	0.6608	1.334	0.1841	intergenic	NA	0.259	1
rs9305727	42525411	21	G	0.2676	0.2006	-0.1255	0.6608	1.334	0.1841	intergenic	NA	0.379	1
rs9305728	42525448	21	A	0.2676	0.2006	-0.1255	0.6608	1.334	0.1841	intergenic	NA	8.162	1
rs9980363	42520134	21	C	0.2659	0.2005	-0.1271	0.659	1.326	0.1868	promoter	LINC00323	3.326	1
rs9980363	42520134	21	C	0.2659	0.2005	-0.1271	0.659	1.326	0.1868	intergenic	NA	3.326	1
rs11701197	42522788	21	A	0.2665	0.201	-0.1275	0.6604	1.326	0.187	intergenic	NA	2.388	1
rs11701356	42522963	21	C	0.2643	0.1999	-0.1275	0.6562	1.322	0.188	intergenic	NA	1.832	1
rs10154102	42523427	21	G	0.2643	0.1999	-0.1275	0.6562	1.322	0.188	intergenic	NA	1.302	1
rs10775668	42678029	21	T	0.3202	0.2423	-0.1547	0.7951	1.321	0.1883	intron	FAM3B	5.034	1
rs9976125	42678988	21	G	0.3202	0.2423	-0.1547	0.7951	1.321	0.1883	intron	FAM3B	0.473	1
rs9976433	42679010	21	A	0.3202	0.2423	-0.1547	0.7951	1.321	0.1883	intron	FAM3B	3.661	1
rs9976216	42679037	21	G	0.3202	0.2423	-0.1547	0.7951	1.321	0.1883	intron	FAM3B	1.672	1
rs9976217	42679042	21	G	0.3202	0.2423	-0.1547	0.7951	1.321	0.1883	intron	FAM3B	0.533	1
rs9976965	42679090	21	C	0.3165	0.2421	-0.1581	0.791	1.307	0.1931	intron	FAM3B	0.561	1
rs8134468	42638685	21	A	-0.3205	0.246	-0.8027	0.1616	-1.303	0.1946	intron	BACE2	2.572	1
rs11701489	42517643	21	A	0.2564	0.1973	-0.1303	0.643	1.3	0.1957	intron	LINC00323	0.265	1
rs11702600	42524832	21	G	0.2619	0.2023	-0.1346	0.6584	1.295	0.1975	intergenic	NA	0.534	1
rs9974272	42521711	21	G	0.2581	0.2007	-0.1352	0.6515	1.286	0.2003	promoter	LINC00323	1.91	1
rs9974272	42521711	21	G	0.2581	0.2007	-0.1352	0.6515	1.286	0.2003	intergenic	NA	1.91	1
rs8127156	42543403	21	G	0.2139	0.1665	-0.1125	0.5402	1.284	0.2009	intron	BACE2	4.064	1
rs9982271	42519389	21	T	0.2532	0.198	-0.1349	0.6414	1.279	0.203	intron	LINC00323	5.274	1
rs9984070	42516327	21	T	0.2529	0.1985	-0.1361	0.642	1.274	0.2046	intron	LINC00323	1.813	1
rs2150425	42531759	21	A	0.1826	0.1581	-0.1272	0.4925	1.155	0.2499	intergenic	NA	7.144	1
rs2007397	42516192	21	A	-0.2647	0.2359	-0.727	0.1977	-1.122	0.2637	intron	LINC00323	0.221	1
rs6517653	42534253	21	A	0.3597	0.3396	-0.3059	1.025	1.059	0.2912	intergenic	NA	0.477	1
rs9305731	42598396	21	G	0.3527	0.3356	-0.305	1.01	1.051	0.2949	intron	BACE2	2.492	1
rs2016240	42530260	21	T	0.353	0.3433	-0.3199	1.026	1.028	0.3056	intergenic	NA	0.033	1
rs2838002	42648767	21	C	0.3183	0.3169	-0.3028	0.9394	1.005	0.3168	intergenic	NA	2.123	1
rs2838003	42649357	21	C	0.3183	0.3169	-0.3028	0.9394	1.005	0.3168	intergenic	NA	3.146	1
rs11700917	42517222	21	T	0.3457	0.3456	-0.3317	1.023	1	0.3188	intron	LINC00323	2.414	1
rs9982181	42599843	21	T	0.3326	0.3443	-0.3423	1.008	0.966	0.3356	intron	BACE2	3.303	1
rs8133778	42642038	21	A	0.3065	0.3182	-0.3172	0.9301	0.9631	0.3371	intron	BACE2	6.941	1
rs55853145	42524465	21	A	0.3014	0.3165	-0.3189	0.9218	0.9523	0.3425	intergenic	NA	3.721	1
rs67757006	42525663	21	A	0.3014	0.3165	-0.3189	0.9218	0.9523	0.3425	intergenic	NA	0.396	1
rs57911156	42522469	21	C	0.3014	0.3175	-0.321	0.9238	0.9492	0.3441	intergenic	NA	1.935	1

rs35335714	42517341	21	C	0.2066	0.2202	-0.225	0.6383	0.9383	0.3496	intron	LINC00323	0.889	1
rs62217917	42527354	21	A	0.2988	0.3188	-0.326	0.9236	0.9372	0.3502	intergenic	NA	3.01	1
rs7278856	42598887	21	G	0.2794	0.3147	-0.3375	0.8962	0.8877	0.3762	intron	BACE2	4.367	1
rs62217923	42539293	21	G	0.1443	0.165	-0.1791	0.4678	0.8747	0.3832	promoter	MIR3197	7.924	1
rs62217923	42539293	21	G	0.1443	0.165	-0.1791	0.4678	0.8747	0.3832	promoter	BACE2	7.924	1
rs62217923	42539293	21	G	0.1443	0.165	-0.1791	0.4678	0.8747	0.3832	intergenic	NA	7.924	1
rs8134160	42541202	21	T	0.1442	0.1657	-0.1807	0.469	0.8699	0.3858	intron	BACE2	4.229	1
rs2837971	42601214	21	A	0.2716	0.3183	-0.3522	0.8954	0.8534	0.3948	intron	BACE2	0.629	1
rs2297271	42520327	21	G	-0.1442	0.1763	-0.4897	0.2013	-0.8179	0.4147	promoter	LINC00323	1.617	1
rs2297271	42520327	21	G	-0.1442	0.1763	-0.4897	0.2013	-0.8179	0.4147	intergenic	NA	1.617	1
rs2248555	42520337	21	G	-0.1442	0.1763	-0.4897	0.2013	-0.8179	0.4147	promoter	LINC00323	3.998	1
rs2248555	42520337	21	G	-0.1442	0.1763	-0.4897	0.2013	-0.8179	0.4147	intergenic	NA	3.998	1
rs9978431	42645737	21	T	0.1402	0.1734	-0.1998	0.4801	0.8081	0.4203	intron	BACE2	4.656	1
rs914174	42517678	21	A	0.1392	0.1744	-0.2026	0.4809	0.7981	0.4261	intron	LINC00323	1.085	1
rs9983938	42516357	21	G	0.1367	0.1758	-0.2079	0.4813	0.7773	0.4382	intron	LINC00323	2.264	1
rs7276900	42532075	21	A	0.1259	0.1645	-0.1965	0.4484	0.7655	0.4451	intergenic	NA	6.65	1
rs880370	42527504	21	A	0.1178	0.1546	-0.1852	0.4207	0.7619	0.4473	intergenic	NA	1.182	1
rs876817	42528499	21	C	0.1248	0.1644	-0.1975	0.447	0.7588	0.4492	intergenic	NA	4.94	1
rs1077117	42599364	21	G	0.2352	0.3164	-0.385	0.8553	0.7433	0.4585	intron	BACE2	4.443	1
rs2837961	42518599	21	C	-0.1279	0.1762	-0.4732	0.2173	-0.7261	0.4689	intron	LINC00323	0.567	1
rs2837990	42620149	21	A	0.2274	0.3199	-0.3996	0.8545	0.7109	0.4782	intron	BACE2	4.605	1
rs2837991	42620160	21	A	0.2274	0.3199	-0.3996	0.8545	0.7109	0.4782	intron	BACE2	3.068	1
rs12627075	42520443	21	C	-0.1248	0.1761	-0.47	0.2205	-0.7083	0.4799	promoter	LINC00323	1.452	1
rs12627075	42520443	21	C	-0.1248	0.1761	-0.47	0.2205	-0.7083	0.4799	intergenic	NA	1.452	1
rs2210277	42536308	21	C	0.1143	0.1631	-0.2054	0.4341	0.7009	0.4844	intergenic	NA	1.445	1
rs1810868	42651481	21	A	-0.1049	0.1525	-0.4038	0.194	-0.6879	0.4926	intergenic	NA	0.102	1
rs28360666	42517239	21	T	-0.1198	0.1751	-0.4629	0.2234	-0.6841	0.4949	intron	LINC00323	0.431	1
rs2837989	42620119	21	A	0.2176	0.3235	-0.4165	0.8517	0.6726	0.5022	intron	BACE2	5.888	1
rs67525224	42627220	21	C	0.2158	0.3227	-0.4167	0.8484	0.6687	0.5047	intron	BACE2	0.663	1
rs11702001	42627969	21	A	0.2158	0.3227	-0.4167	0.8484	0.6687	0.5047	intron	BACE2	1.969	1
rs9980188	42664366	21	G	0.09576	0.1441	-0.1866	0.3781	0.6646	0.5073	intergenic	NA	0.133	1
rs13049769	42664689	21	C	0.09576	0.1441	-0.1866	0.3781	0.6646	0.5073	intergenic	NA	0.243	1
rs7279273	42664100	21	C	0.09587	0.1451	-0.1885	0.3802	0.6607	0.5098	intergenic	NA	3.185	1
rs12482242	42648821	21	G	-0.09534	0.1527	-0.3946	0.2039	-0.6244	0.5333	intergenic	NA	5.968	1
rs2838004	42650654	21	G	-0.09534	0.1527	-0.3946	0.2039	-0.6244	0.5333	intergenic	NA	11.16	1
rs9305733	42642699	21	C	-0.09544	0.1543	-0.3979	0.207	-0.6184	0.5373	intron	BACE2	1.263	1
rs8127120	42619274	21	G	0.2052	0.3344	-0.4503	0.8606	0.6135	0.5405	intron	BACE2	4.441	1
rs9976426	42641913	21	T	-0.09406	0.155	-0.3979	0.2098	-0.6068	0.5449	intron	BACE2	1.811	1
rs11700778	42517399	21	A	0.2021	0.3341	-0.4527	0.8569	0.605	0.5461	intron	LINC00323	1.427	1
rs2898440	42517480	21	A	0.2021	0.3341	-0.4527	0.8569	0.605	0.5461	intron	LINC00323	1.485	1
rs914173	42517552	21	C	0.2021	0.3341	-0.4527	0.8569	0.605	0.5461	intron	LINC00323	1.058	1
rs914175	42517750	21	A	0.2021	0.3341	-0.4527	0.8569	0.605	0.5461	intron	LINC00323	2.443	1
rs7281814	42518444	21	G	0.2021	0.3341	-0.4527	0.8569	0.605	0.5461	intron	LINC00323	4.384	1
rs2837962	42518650	21	G	0.2021	0.3341	-0.4527	0.8569	0.605	0.5461	intron	LINC00323	9.818	1
rs8134992	42624622	21	T	0.1872	0.333	-0.4655	0.8399	0.5622	0.5748	intron	BACE2	0.409	1
rs1041445	42515737	21	T	0.1713	0.312	-0.4401	0.7828	0.5492	0.5837	intron	LINC00323	0.89	1
rs2837994	42624124	21	A	0.1816	0.3323	-0.4696	0.8328	0.5465	0.5855	intron	BACE2	2.04	1
rs6517655	42536246	21	C	0.09211	0.1735	-0.248	0.4322	0.5308	0.5964	intergenic	NA	0.17	1
rs58867243	42644663	21	A	-0.1684	0.3262	-0.8076	0.4709	-0.5162	0.6064	intron	BACE2	11.57	1
rs12483323	42643845	21	T	-0.1221	0.2586	-0.6289	0.3847	-0.4723	0.6374	intron	BACE2	2.526	1
rs2776342	42521213	21	C	0.1179	0.3189	-0.507	0.7429	0.3699	0.712	promoter	LINC00323	2.557	1
rs2776342	42521213	21	C	0.1179	0.3189	-0.507	0.7429	0.3699	0.712	intergenic	NA	2.557	1
rs2776343	42521267	21	A	0.1179	0.3189	-0.507	0.7429	0.3699	0.712	promoter	LINC00323	0.128	1
rs2776343	42521267	21	A	0.1179	0.3189	-0.507	0.7429	0.3699	0.712	intergenic	NA	0.128	1
rs2705572	42521161	21	G	0.1172	0.318	-0.5062	0.7406	0.3685	0.713	promoter	LINC00323	0.182	1
rs2705572	42521161	21	G	0.1172	0.318	-0.5062	0.7406	0.3685	0.713	intergenic	NA	0.182	1
rs11088544	42517484	21	C	0.1168	0.3173	-0.5052	0.7387	0.3679	0.7134	intron	LINC00323	0.242	1
rs1571732	42517559	21	G	0.111	0.3172	-0.5107	0.7326	0.3499	0.7269	intron	LINC00323	0.225	1

rs914176	42528192	21	G	0.05793	0.1722	-0.2796	0.3954	0.3364	0.737	intergenic	NA	0.126	1
rs2837964	42520646	21	A	0.1053	0.3156	-0.5132	0.7239	0.3337	0.7391	promoter	LINC00323	1.212	1
rs2837964	42520646	21	A	0.1053	0.3156	-0.5132	0.7239	0.3337	0.7391	intergenic	NA	1.212	1
rs9981553	42620680	21	T	0.05503	0.1698	-0.2778	0.3878	0.3241	0.7463	intron	BACE2	2.454	1
rs11909506	42678742	21	G	0.05724	0.2021	-0.3388	0.4533	0.2833	0.7774	intron	FAM3B	0.63	1
rs12482462	42626851	21	T	0.04689	0.1695	-0.2854	0.3792	0.2766	0.7825	intron	BACE2	11.02	1
rs2837996	42626706	21	T	-0.02996	0.2046	-0.431	0.3711	-0.1464	0.8838	intron	BACE2	0.264	1
rs2837993	42620714	21	T	0.02426	0.1713	-0.3114	0.36	0.1416	0.8876	intron	BACE2	0.306	1
rs2837999	42630531	21	A	-0.009054	0.201	-0.4029	0.3848	-0.04505	0.9641	intron	BACE2	1.506	1
rs13048452	42532909	21	G	-0.005133	0.1617	-0.3221	0.3118	-0.03174	0.9747	intergenic	NA	1.938	1
rs11088546	42532977	21	A	-0.005133	0.1617	-0.3221	0.3118	-0.03174	0.9747	intergenic	NA	2.54	1
rs960231	42622657	21	G	-0.004926	0.2019	-0.4006	0.3907	-0.0244	0.9806	intron	BACE2	3.465	1
rs7279020	42534031	21	T	0.003743	0.1628	-0.3153	0.3227	0.023	0.9817	intergenic	NA	1.664	1
rs9975388	42626362	21	C	-0.002941	0.2017	-0.3983	0.3925	-0.01458	0.9884	intron	BACE2	4.067	1
rs2183588	42626882	21	A	-0.001865	0.1988	-0.3916	0.3878	-0.009381	0.9925	intron	BACE2	1.744	1
rs57576269	42516902	21	A	NA	NA	NA	NA	NA	NA	intron	LINC00323	2.078	1
rs1571733	42518034	21	A	NA	NA	NA	NA	NA	NA	intron	LINC00323	3.33	1
rs62217915	42519037	21	C	NA	NA	NA	NA	NA	NA	intron	LINC00323	1.443	1
rs73362453	42519604	21	T	NA	NA	NA	NA	NA	NA	intron	LINC00323	0.094	1
rs74594237	42529023	21	A	NA	NA	NA	NA	NA	NA	intergenic	NA	0.546	1
rs2410404	42529908	21	T	NA	NA	NA	NA	NA	NA	intergenic	NA	0.078	1
rs77270082	42531725	21	T	NA	NA	NA	NA	NA	NA	intergenic	NA	3.295	1
rs62217922	42532753	21	A	NA	NA	NA	NA	NA	NA	intergenic	NA	0.959	1
rs3787933	42573589	21	G	NA	NA	NA	NA	NA	NA	intron	BACE2	0.916	1
rs4816714	42574691	21	G	NA	NA	NA	NA	NA	NA	intron	BACE2	2.961	1
rs4818222	42574696	21	G	NA	NA	NA	NA	NA	NA	intron	BACE2	4.834	1
rs7277920	42575415	21	C	NA	NA	NA	NA	NA	NA	intron	BACE2	0.081	1
rs73364465	42577778	21	G	NA	NA	NA	NA	NA	NA	intron	BACE2	0.367	1
rs75762331	42581079	21	A	NA	NA	NA	NA	NA	NA	intron	BACE2	1.284	1
rs34019132	42583154	21	T	NA	NA	NA	NA	NA	NA	intron	BACE2	1.863	1
rs6517656	42583738	21	A	NA	NA	NA	NA	NA	NA	intron	BACE2	15.65	1
rs28360503	42583804	21	C	NA	NA	NA	NA	NA	NA	intron	BACE2	1.192	1
rs6517657	42584141	21	T	NA	NA	NA	NA	NA	NA	intron	BACE2	1.966	1
rs35939063	42584621	21	G	NA	NA	NA	NA	NA	NA	intron	BACE2	4.171	1
rs13052926	42585088	21	G	NA	NA	NA	NA	NA	NA	intron	BACE2	1.269	1
rs2837969	42588377	21	T	NA	NA	NA	NA	NA	NA	intron	BACE2	1.183	1
rs10451740	42588731	21	C	NA	NA	NA	NA	NA	NA	intron	BACE2	1.848	1
rs80303768	42594678	21	A	NA	NA	NA	NA	NA	NA	intron	BACE2	4.279	1
rs28629220	42597387	21	C	NA	NA	NA	NA	NA	NA	intron	BACE2	3.563	1
rs17000688	42599218	21	A	NA	NA	NA	NA	NA	NA	intron	BACE2	4.117	1
rs12627301	42599787	21	C	NA	NA	NA	NA	NA	NA	intron	BACE2	0.684	1
rs59760478	42600934	21	A	NA	NA	NA	NA	NA	NA	intron	BACE2	1.333	1
rs2837972	42601741	21	G	NA	NA	NA	NA	NA	NA	intron	BACE2	1.213	1
rs2837973	42602088	21	G	NA	NA	NA	NA	NA	NA	intron	BACE2	3.235	1
rs2837974	42602735	21	G	NA	NA	NA	NA	NA	NA	intron	BACE2	1.655	1
rs142329681	42619591	21	A	NA	NA	NA	NA	NA	NA	intron	BACE2	3.299	1
rs75303510	42620353	21	A	NA	NA	NA	NA	NA	NA	intron	BACE2	0.518	1
rs960230	42622479	21	A	NA	NA	NA	NA	NA	NA	intron	BACE2	0.443	1
rs2837995	42624470	21	T	NA	NA	NA	NA	NA	NA	intron	BACE2	5.871	1
rs77395637	42627316	21	A	NA	NA	NA	NA	NA	NA	intron	BACE2	2.997	1
rs3787944	42629744	21	T	NA	NA	NA	NA	NA	NA	intron	BACE2	1.453	1
rs75748434	42630703	21	T	NA	NA	NA	NA	NA	NA	intron	BACE2	2.631	1
rs67837204	42632585	21	T	NA	NA	NA	NA	NA	NA	intron	BACE2	2.816	1
rs76228446	42632720	21	T	NA	NA	NA	NA	NA	NA	intron	BACE2	0.535	1
rs11701756	42632770	21	A	NA	NA	NA	NA	NA	NA	intron	BACE2	0.192	1
rs10483075	42633268	21	C	NA	NA	NA	NA	NA	NA	intron	BACE2	1.747	1
rs62219576	42633690	21	A	NA	NA	NA	NA	NA	NA	intron	BACE2	5.731	1
rs59836194	42634841	21	T	NA	NA	NA	NA	NA	NA	intron	BACE2	1.754	1

rs726980	42635676	21	A	NA	NA	NA	NA	NA	NA	intron	BACE2	0.938	1
rs74477222	42636501	21	T	NA	NA	NA	NA	NA	NA	intron	BACE2	1.786	1
rs7277675	42637222	21	T	NA	NA	NA	NA	NA	NA	intron	BACE2	0.433	1
rs58404061	42638931	21	A	NA	NA	NA	NA	NA	NA	intron	BACE2	1.743	1
rs3787945	42640022	21	T	NA	NA	NA	NA	NA	NA	intron	BACE2	1.743	1
rs75306103	42640410	21	A	NA	NA	NA	NA	NA	NA	intron	BACE2	0.758	1
rs58534150	42640903	21	T	NA	NA	NA	NA	NA	NA	intron	BACE2	0.04	1
rs6517659	42641982	21	T	NA	NA	NA	NA	NA	NA	intron	BACE2	2.702	1
rs68087522	42642096	21	G	NA	NA	NA	NA	NA	NA	intron	BACE2	5.155	1
rs77240271	42642405	21	A	NA	NA	NA	NA	NA	NA	intron	BACE2	2.167	1
rs9808711	42642642	21	A	NA	NA	NA	NA	NA	NA	intron	BACE2	2.893	1
rs58003932	42642825	21	G	NA	NA	NA	NA	NA	NA	intron	BACE2	5.921	1
rs62219579	42643610	21	C	NA	NA	NA	NA	NA	NA	intron	BACE2	1.035	1
rs4818228	42643772	21	A	NA	NA	NA	NA	NA	NA	intron	BACE2	2.695	1
rs4818229	42643968	21	T	NA	NA	NA	NA	NA	NA	intron	BACE2	3.003	1
rs75692138	42644452	21	A	NA	NA	NA	NA	NA	NA	intron	BACE2	3.888	1
rs75007172	42644515	21	A	NA	NA	NA	NA	NA	NA	intron	BACE2	5.027	1
rs34996007	42644885	21	T	NA	NA	NA	NA	NA	NA	intron	BACE2	9.399	1
rs11700718	42644982	21	C	NA	NA	NA	NA	NA	NA	intron	BACE2	2.431	1
rs61326441	42645938	21	A	NA	NA	NA	NA	NA	NA	intron	BACE2	1.147	1
rs2838000	42646518	21	A	NA	NA	NA	NA	NA	NA	intron	BACE2	0.091	1
rs2838001	42646992	21	C	NA	NA	NA	NA	NA	NA	intron	BACE2	0.276	1
rs62219580	42649211	21	G	NA	NA	NA	NA	NA	NA	intergenic	NA	2.516	1
rs73224110	42650666	21	A	NA	NA	NA	NA	NA	NA	intergenic	NA	0.295	1
rs11701157	42651480	21	A	NA	NA	NA	NA	NA	NA	intergenic	NA	0.139	1
rs111511447	42651495	21	A	NA	NA	NA	NA	NA	NA	intergenic	NA	0.654	1
rs3746889	42651946	21	C	NA	NA	NA	NA	NA	NA	intergenic	NA	0.381	1
rs11559045	42652831	21	T	NA	NA	NA	NA	NA	NA	intergenic	NA	0.346	1
rs117606823	42653122	21	G	NA	NA	NA	NA	NA	NA	intergenic	NA	4.62	1
rs73224118	42653246	21	T	NA	NA	NA	NA	NA	NA	intergenic	NA	9.325	1
rs1999331	42654497	21	A	NA	NA	NA	NA	NA	NA	intergenic	NA	0.914	1
rs75000682	42662479	21	A	NA	NA	NA	NA	NA	NA	intergenic	NA	2.753	1
rs73905312	42663603	21	A	NA	NA	NA	NA	NA	NA	intergenic	NA	12.5	1
rs73905316	42668059	21	T	NA	NA	NA	NA	NA	NA	intergenic	NA	0.853	1
rs67438118	42669465	21	A	NA	NA	NA	NA	NA	NA	intergenic	NA	0.185	1
rs76981906	42669740	21	A	NA	NA	NA	NA	NA	NA	intergenic	NA	3.008	1
rs7276719	42670165	21	A	NA	NA	NA	NA	NA	NA	intergenic	NA	6.32	1
rs146048697	42672680	21	T	NA	NA	NA	NA	NA	NA	intergenic	NA	6.555	1
rs4816716	42674021	21	A	NA	NA	NA	NA	NA	NA	intergenic	NA	0.667	1
rs6517662	42674022	21	A	NA	NA	NA	NA	NA	NA	intergenic	NA	0.726	1
rs75896199	42678194	21	G	NA	NA	NA	NA	NA	NA	intron	FAM3B	3.138	1
rs57347504	42678481	21	G	NA	NA	NA	NA	NA	NA	intron	FAM3B	0.926	1
rs68183718	42678791	21	T	NA	NA	NA	NA	NA	NA	intron	FAM3B	0.914	1

Supplementary Table 8

SNP	BP	CHR	A1	BETA	SE	L95	U95	STAT	P	LOCATION	GENESYMBOL	CADD	AdjP
rs2410415	42641402	21	C	-0.3243	0.1044	-0.529	-0.1196	-3.106	0.002126	intron	BACE2	5.85	0.091418
rs8134468	42638685	21	A	-0.324	0.1045	-0.5288	-0.1192	-3.101	0.002158	intron	BACE2	2.572	0.092794
rs11700917	42517222	21	T	0.2969	0.1594	-0.01554	0.6094	1.862	0.06375	intron	LINC00323	2.414	1
rs11700778	42517399	21	A	0.2691	0.1595	-0.04359	0.5818	1.687	0.09292	intron	LINC00323	1.427	1
rs2016240	42530260	21	T	0.258	0.1545	-0.04468	0.5608	1.671	0.09607	intergenic	NA	0.033	1
rs2898440	42517480	21	A	0.2658	0.1608	-0.04928	0.581	1.653	0.0995	intron	LINC00323	1.485	1
rs914173	42517552	21	C	0.2658	0.1608	-0.04928	0.581	1.653	0.0995	intron	LINC00323	1.058	1
rs914175	42517750	21	A	0.2658	0.1608	-0.04928	0.581	1.653	0.0995	intron	LINC00323	2.443	1
rs7281814	42518444	21	G	0.2658	0.1608	-0.04928	0.581	1.653	0.0995	intron	LINC00323	4.384	1
rs2837962	42518650	21	G	0.2658	0.1608	-0.04928	0.581	1.653	0.0995	intron	LINC00323	9.818	1
rs6517653	42534253	21	A	0.255	0.1582	-0.05505	0.565	1.612	0.1082	intergenic	NA	0.477	1
rs11702409	42537012	21	G	0.2565	0.1591	-0.05533	0.5683	1.612	0.1082	intergenic	NA	0.788	1
rs7277920	42575415	21	C	0.3146	0.2072	-0.09154	0.7208	1.518	0.1303	intron	BACE2	0.081	1
rs4816714	42574691	21	G	0.3128	0.2075	-0.09381	0.7194	1.508	0.1329	intron	BACE2	2.961	1
rs4818222	42574696	21	G	0.3128	0.2075	-0.09381	0.7194	1.508	0.1329	intron	BACE2	4.834	1
rs3787933	42573589	21	G	0.2681	0.1803	-0.08526	0.6215	1.487	0.1383	intron	BACE2	0.916	1
rs13052926	42585088	21	G	0.2538	0.1781	-0.09534	0.603	1.425	0.1555	intron	BACE2	1.269	1
rs35939063	42584621	21	G	0.2531	0.1799	-0.09949	0.6056	1.407	0.1608	intron	BACE2	4.171	1
rs2410404	42529908	21	T	-0.2534	0.2065	-0.658	0.1513	-1.227	0.2209	intergenic	NA	0.078	1
rs77270082	42531725	21	T	-0.2534	0.2065	-0.658	0.1513	-1.227	0.2209	intergenic	NA	3.295	1
rs2837964	42520646	21	A	0.119	0.09708	-0.07126	0.3093	1.226	0.2214	promoter	LINC00323	1.212	1
rs2837964	42520646	21	A	0.119	0.09708	-0.07126	0.3093	1.226	0.2214	intergenic	NA	1.212	1
rs74594237	42529023	21	A	-0.2525	0.2072	-0.6587	0.1537	-1.218	0.2242	intergenic	NA	0.546	1
rs62217922	42532753	21	A	-0.2525	0.2072	-0.6587	0.1537	-1.218	0.2242	intergenic	NA	0.959	1
rs1041445	42515737	21	T	0.119	0.09812	-0.07329	0.3113	1.213	0.2263	intron	LINC00323	0.89	1
rs11088544	42517484	21	C	0.1081	0.09559	-0.07923	0.2955	1.131	0.2591	intron	LINC00323	0.242	1
rs1571732	42517559	21	G	0.1084	0.09593	-0.07961	0.2964	1.13	0.2596	intron	LINC00323	0.225	1
rs2705572	42521161	21	G	0.1067	0.09602	-0.0815	0.2949	1.111	0.2676	promoter	LINC00323	0.182	1
rs2705572	42521161	21	G	0.1067	0.09602	-0.0815	0.2949	1.111	0.2676	intergenic	NA	0.182	1
rs2776342	42521213	21	C	0.1068	0.09619	-0.08169	0.2954	1.111	0.2678	promoter	LINC00323	2.557	1
rs2776342	42521213	21	C	0.1068	0.09619	-0.08169	0.2954	1.111	0.2678	intergenic	NA	2.557	1
rs2776343	42521267	21	A	0.1068	0.09619	-0.08169	0.2954	1.111	0.2678	promoter	LINC00323	0.128	1
rs2776343	42521267	21	A	0.1068	0.09619	-0.08169	0.2954	1.111	0.2678	intergenic	NA	0.128	1
rs9978431	42645737	21	T	0.0656	0.06294	-0.05775	0.189	1.042	0.2983	intron	BACE2	4.656	1
rs7278856	42598887	21	G	-0.155	0.1552	-0.4592	0.1492	-0.9988	0.3189	intron	BACE2	4.367	1
rs12483323	42643845	21	T	-0.1559	0.1616	-0.4726	0.1608	-0.965	0.3355	intron	BACE2	2.526	1
rs1571733	42518034	21	A	-0.1648	0.1803	-0.5183	0.1886	-0.9141	0.3616	intron	LINC00323	3.33	1
rs1077117	42599364	21	G	-0.1418	0.1575	-0.4505	0.167	-0.8999	0.3691	intron	BACE2	4.443	1
rs2210277	42536308	21	C	-0.06183	0.0708	-0.2006	0.07694	-0.8733	0.3833	intergenic	NA	1.445	1
rs2837971	42601214	21	A	-0.1373	0.1601	-0.4511	0.1766	-0.8572	0.3922	intron	BACE2	0.629	1

rs62217915	42519037	21	C	-0.1664	0.207	-0.5721	0.2392	-0.8042	0.4221	intron	LINC00323	1.443	1
rs880370	42527504	21	A	0.04372	0.05541	-0.06488	0.1523	0.7891	0.4309	intergenic	NA	1.182	1
rs2838002	42648767	21	C	0.08607	0.1095	-0.1286	0.3007	0.7858	0.4328	intergenic	NA	2.123	1
rs2838003	42649357	21	C	0.08607	0.1095	-0.1286	0.3007	0.7858	0.4328	intergenic	NA	3.146	1
rs7276900	42532075	21	A	-0.05554	0.07091	-0.1945	0.08344	-0.7832	0.4342	intergenic	NA	6.65	1
rs6517655	42536246	21	C	-0.05457	0.07084	-0.1934	0.08427	-0.7703	0.4418	intergenic	NA	0.17	1
rs7279273	42664100	21	C	0.04579	0.06099	-0.07376	0.1653	0.7507	0.4536	intergenic	NA	3.185	1
rs6517657	42584141	21	T	0.1068	0.1469	-0.1811	0.3946	0.727	0.4679	intron	BACE2	1.966	1
rs34019132	42583154	21	T	0.1067	0.1478	-0.183	0.3964	0.7219	0.471	intron	BACE2	1.863	1
rs28360503	42583804	21	C	0.1067	0.1481	-0.1836	0.397	0.7203	0.472	intron	BACE2	1.192	1
rs9980188	42664366	21	G	0.04298	0.06069	-0.07596	0.1619	0.7082	0.4795	intergenic	NA	0.133	1
rs13049769	42664689	21	C	0.04298	0.06069	-0.07596	0.1619	0.7082	0.4795	intergenic	NA	0.243	1
rs55853145	42524465	21	A	-0.07879	0.1116	-0.2975	0.1399	-0.7062	0.4807	intergenic	NA	3.721	1
rs67757006	42525663	21	A	-0.07879	0.1116	-0.2975	0.1399	-0.7062	0.4807	intergenic	NA	0.396	1
rs57911156	42522469	21	C	-0.07883	0.1118	-0.298	0.1403	-0.7051	0.4814	intergenic	NA	1.935	1
rs6517656	42583738	21	A	0.1039	0.1481	-0.1863	0.3941	0.7018	0.4835	intron	BACE2	15.65	1
rs876817	42528499	21	C	-0.0472	0.07427	-0.1928	0.09836	-0.6355	0.5257	intergenic	NA	4.94	1
rs11700578	42522350	21	C	0.04761	0.07524	-0.09985	0.1951	0.6328	0.5275	intergenic	NA	1.044	1
rs11700757	42522447	21	T	0.04761	0.07524	-0.09985	0.1951	0.6328	0.5275	intergenic	NA	0.072	1
rs11700599	42522499	21	C	0.04761	0.07524	-0.09985	0.1951	0.6328	0.5275	intergenic	NA	8.106	1
rs11701114	42522656	21	A	0.04761	0.07524	-0.09985	0.1951	0.6328	0.5275	intergenic	NA	0.019	1
rs8133778	42642038	21	A	0.05597	0.09428	-0.1288	0.2408	0.5936	0.5533	intron	BACE2	6.941	1
rs62217917	42527354	21	A	-0.06405	0.1083	-0.2763	0.1482	-0.5915	0.5548	intergenic	NA	3.01	1
rs2183588	42626882	21	A	0.04131	0.07772	-0.111	0.1936	0.5316	0.5955	intron	BACE2	1.744	1
rs9975388	42626362	21	C	0.04083	0.07789	-0.1118	0.1935	0.5242	0.6006	intron	BACE2	4.067	1
rs2012050	42651562	21	T	0.03771	0.07558	-0.1104	0.1858	0.4989	0.6183	intergenic	NA	4.184	1
rs6517654	42534328	21	C	-0.0424	0.0852	-0.2094	0.1246	-0.4977	0.6192	intergenic	NA	0.441	1
rs960231	42622657	21	G	0.03849	0.07805	-0.1145	0.1915	0.4932	0.6223	intron	BACE2	3.465	1
rs2837999	42630531	21	A	0.04279	0.08707	-0.1279	0.2134	0.4914	0.6236	intron	BACE2	1.506	1
rs2838001	42646992	21	C	-0.07477	0.1587	-0.3859	0.2364	-0.471	0.6381	intron	BACE2	0.276	1
rs2838000	42646518	21	A	-0.07444	0.1589	-0.3859	0.2371	-0.4684	0.6399	intron	BACE2	0.091	1
rs28654619	42536126	21	C	-0.04055	0.08881	-0.2146	0.1335	-0.4567	0.6483	intergenic	NA	3.37	1
rs62217923	42539293	21	G	-0.0348	0.07716	-0.186	0.1164	-0.451	0.6524	promoter	MIR3197	7.924	1
rs62217923	42539293	21	G	-0.0348	0.07716	-0.186	0.1164	-0.451	0.6524	promoter	BACE2	7.924	1
rs62217923	42539293	21	G	-0.0348	0.07716	-0.186	0.1164	-0.451	0.6524	intergenic	NA	7.924	1
rs6517659	42641982	21	T	-0.06799	0.1619	-0.3853	0.2493	-0.42	0.6749	intron	BACE2	2.702	1
rs9983938	42516357	21	G	-0.03463	0.08442	-0.2001	0.1308	-0.4102	0.682	intron	LINC00323	2.264	1
rs2150425	42531759	21	A	0.02468	0.06017	-0.09325	0.1426	0.4102	0.682	intergenic	NA	7.144	1
rs1810868	42651481	21	A	0.02456	0.06095	-0.0949	0.144	0.4029	0.6874	intergenic	NA	0.102	1
rs9975636	42531333	21	A	-0.03511	0.08877	-0.2091	0.1389	-0.3956	0.6927	intergenic	NA	1.541	1
rs914174	42517678	21	A	-0.03265	0.08413	-0.1975	0.1322	-0.388	0.6983	intron	LINC00323	1.085	1
rs9981553	42620680	21	T	0.02064	0.05755	-0.09216	0.1334	0.3587	0.7201	intron	BACE2	2.454	1
rs12482462	42626851	21	T	0.02024	0.05746	-0.09237	0.1329	0.3523	0.7249	intron	BACE2	11.02	1

rs12482242	42648821	21	G	0.0204	0.05926	-0.09575	0.1365	0.3442	0.731	intergenic	NA	5.968	1
rs2838004	42650654	21	G	0.0204	0.05926	-0.09575	0.1365	0.3442	0.731	intergenic	NA	11.16	1
rs12627075	42520443	21	C	-0.02042	0.0621	-0.1421	0.1013	-0.3288	0.7426	promoter	LINC00323	1.452	1
rs12627075	42520443	21	C	-0.02042	0.0621	-0.1421	0.1013	-0.3288	0.7426	intergenic	NA	1.452	1
rs9976426	42641913	21	T	0.01789	0.05942	-0.09858	0.1344	0.301	0.7637	intron	BACE2	1.811	1
rs9305733	42642699	21	C	0.01789	0.05942	-0.09858	0.1344	0.301	0.7637	intron	BACE2	1.263	1
rs8134160	42541202	21	T	-0.02323	0.07923	-0.1785	0.1321	-0.2932	0.7696	intron	BACE2	4.229	1
rs2837990	42620149	21	A	-0.04513	0.1562	-0.3514	0.2611	-0.2888	0.773	intron	BACE2	4.605	1
rs2837991	42620160	21	A	-0.04513	0.1562	-0.3514	0.2611	-0.2888	0.773	intron	BACE2	3.068	1
rs8127156	42543403	21	G	-0.02258	0.07849	-0.1764	0.1313	-0.2876	0.7739	intron	BACE2	4.064	1
rs4818228	42643772	21	A	-0.04217	0.1614	-0.3585	0.2741	-0.2613	0.7941	intron	BACE2	2.695	1
rs9808711	42642642	21	A	-0.04027	0.1609	-0.3556	0.2751	-0.2503	0.8026	intron	BACE2	2.893	1
rs4818229	42643968	21	T	-0.04027	0.1609	-0.3556	0.2751	-0.2503	0.8026	intron	BACE2	3.003	1
rs9984070	42516327	21	T	-0.02366	0.09789	-0.2155	0.1682	-0.2417	0.8092	intron	LINC00323	1.813	1
rs9982271	42519389	21	T	-0.02276	0.095	-0.209	0.1634	-0.2395	0.8109	intron	LINC00323	5.274	1
rs2297271	42520327	21	G	-0.01392	0.0621	-0.1356	0.1078	-0.2241	0.8229	promoter	LINC00323	1.617	1
rs2297271	42520327	21	G	-0.01392	0.0621	-0.1356	0.1078	-0.2241	0.8229	intergenic	NA	1.617	1
rs2248555	42520337	21	G	-0.01392	0.0621	-0.1356	0.1078	-0.2241	0.8229	promoter	LINC00323	3.998	1
rs2248555	42520337	21	G	-0.01392	0.0621	-0.1356	0.1078	-0.2241	0.8229	intergenic	NA	3.998	1
rs11701489	42517643	21	A	-0.02182	0.09764	-0.2132	0.1695	-0.2235	0.8233	intron	LINC00323	0.265	1
rs28360666	42517239	21	T	-0.01297	0.05996	-0.1305	0.1045	-0.2164	0.8289	intron	LINC00323	0.431	1
rs9980363	42520134	21	C	-0.02093	0.09892	-0.2148	0.1729	-0.2116	0.8326	promoter	LINC00323	3.326	1
rs9980363	42520134	21	C	-0.02093	0.09892	-0.2148	0.1729	-0.2116	0.8326	intergenic	NA	3.326	1
rs11701197	42522788	21	A	-0.02019	0.09831	-0.2129	0.1725	-0.2054	0.8375	intergenic	NA	2.388	1
rs11702600	42524832	21	G	-0.01818	0.09569	-0.2057	0.1694	-0.19	0.8494	intergenic	NA	0.534	1
rs11701851	42523852	21	T	-0.01742	0.09812	-0.2097	0.1749	-0.1775	0.8592	intergenic	NA	0.831	1
rs11702163	42524513	21	C	-0.01742	0.09812	-0.2097	0.1749	-0.1775	0.8592	intergenic	NA	1.858	1
rs11702590	42524628	21	A	-0.01742	0.09812	-0.2097	0.1749	-0.1775	0.8592	intergenic	NA	6.287	1
rs11702752	42524741	21	C	-0.01742	0.09812	-0.2097	0.1749	-0.1775	0.8592	intergenic	NA	0.789	1
rs11702604	42524870	21	T	-0.01742	0.09812	-0.2097	0.1749	-0.1775	0.8592	intergenic	NA	4.253	1
rs10154234	42525007	21	A	-0.01742	0.09812	-0.2097	0.1749	-0.1775	0.8592	intergenic	NA	3.608	1
rs9305724	42525185	21	G	-0.01742	0.09812	-0.2097	0.1749	-0.1775	0.8592	intergenic	NA	1.61	1
rs9305725	42525257	21	C	-0.01742	0.09812	-0.2097	0.1749	-0.1775	0.8592	intergenic	NA	1.78	1
rs9305726	42525272	21	A	-0.01742	0.09812	-0.2097	0.1749	-0.1775	0.8592	intergenic	NA	0.259	1
rs9305727	42525411	21	G	-0.01742	0.09812	-0.2097	0.1749	-0.1775	0.8592	intergenic	NA	0.379	1
rs9305728	42525448	21	A	-0.01742	0.09812	-0.2097	0.1749	-0.1775	0.8592	intergenic	NA	8.162	1
rs13049454	42526520	21	A	-0.01742	0.09812	-0.2097	0.1749	-0.1775	0.8592	intergenic	NA	2.152	1
rs11701356	42522963	21	C	-0.01704	0.09797	-0.2091	0.175	-0.174	0.862	intergenic	NA	1.832	1
rs10154102	42523427	21	G	-0.01704	0.09797	-0.2091	0.175	-0.174	0.862	intergenic	NA	1.302	1
rs10154104	42523511	21	G	-0.01704	0.09797	-0.2091	0.175	-0.174	0.862	intergenic	NA	1.873	1
rs7279020	42534031	21	T	-0.00971	0.05928	-0.1259	0.1065	-0.1638	0.87	intergenic	NA	1.664	1
rs2007397	42516192	21	A	-0.00995	0.06206	-0.1316	0.1117	-0.1604	0.8727	intron	LINC00323	0.221	1
rs2837993	42620714	21	T	-0.00832	0.05684	-0.1197	0.1031	-0.1463	0.8838	intron	BACE2	0.306	1

rs2837961	42518599	21	C	-0.00861	0.06096	-0.1281	0.1109	-0.1412	0.8878	intron	LINC00323	0.567	1
rs914176	42528192	21	G	-0.00712	0.05896	-0.1227	0.1084	-0.1207	0.904	intergenic	NA	0.126	1
rs13048452	42532909	21	G	-0.00666	0.05923	-0.1227	0.1094	-0.1124	0.9106	intergenic	NA	1.938	1
rs11088546	42532977	21	A	-0.00666	0.05923	-0.1227	0.1094	-0.1124	0.9106	intergenic	NA	2.54	1
rs960230	42622479	21	A	0.0117	0.1141	-0.2119	0.2353	0.1026	0.9184	intron	BACE2	0.443	1
rs35335714	42517341	21	C	-0.01009	0.09852	-0.2032	0.183	-0.1024	0.9185	intron	LINC00323	0.889	1
rs2837996	42626706	21	T	0.008147	0.08145	-0.1515	0.1678	0.1	0.9204	intron	BACE2	0.264	1
rs11909506	42678742	21	G	0.005832	0.07609	-0.1433	0.155	0.07665	0.939	intron	FAM3B	0.63	1
rs9976125	42678988	21	G	-0.00581	0.0887	-0.1797	0.168	-0.06552	0.9478	intron	FAM3B	0.473	1
rs9976433	42679010	21	A	-0.00581	0.0887	-0.1797	0.168	-0.06552	0.9478	intron	FAM3B	3.661	1
rs9976216	42679037	21	G	-0.00581	0.0887	-0.1797	0.168	-0.06552	0.9478	intron	FAM3B	1.672	1
rs9976217	42679042	21	G	-0.00581	0.0887	-0.1797	0.168	-0.06552	0.9478	intron	FAM3B	0.533	1
rs9976965	42679090	21	C	-0.00581	0.0887	-0.1797	0.168	-0.06552	0.9478	intron	FAM3B	0.561	1
rs10775668	42678029	21	T	-0.00561	0.08884	-0.1797	0.1685	-0.06308	0.9498	intron	FAM3B	5.034	1
rs9974272	42521711	21	G	-0.00085	0.1168	-0.2298	0.2281	-0.0073	0.9942	promoter	LINC00323	1.91	1
rs9974272	42521711	21	G	-0.00085	0.1168	-0.2298	0.2281	-0.0073	0.9942	intergenic	NA	1.91	1
rs57576269	42516902	21	A	NA	NA	NA	NA	NA	NA	intron	LINC00323	2.078	1
rs73362453	42519604	21	T	NA	NA	NA	NA	NA	NA	intron	LINC00323	0.094	1
rs73364465	42577778	21	G	NA	NA	NA	NA	NA	NA	intron	BACE2	0.367	1
rs75762331	42581079	21	A	NA	NA	NA	NA	NA	NA	intron	BACE2	1.284	1
rs2837969	42588377	21	T	NA	NA	NA	NA	NA	NA	intron	BACE2	1.183	1
rs10451740	42588731	21	C	NA	NA	NA	NA	NA	NA	intron	BACE2	1.848	1
rs80303768	42594678	21	A	NA	NA	NA	NA	NA	NA	intron	BACE2	4.279	1
rs28629220	42597387	21	C	NA	NA	NA	NA	NA	NA	intron	BACE2	3.563	1
rs9305731	42598396	21	G	NA	NA	NA	NA	NA	NA	intron	BACE2	2.492	1
rs17000688	42599218	21	A	NA	NA	NA	NA	NA	NA	intron	BACE2	4.117	1
rs12627301	42599787	21	C	NA	NA	NA	NA	NA	NA	intron	BACE2	0.684	1
rs9982181	42599843	21	T	NA	NA	NA	NA	NA	NA	intron	BACE2	3.303	1
rs59760478	42600934	21	A	NA	NA	NA	NA	NA	NA	intron	BACE2	1.333	1
rs2837972	42601741	21	G	NA	NA	NA	NA	NA	NA	intron	BACE2	1.213	1
rs2837973	42602088	21	G	NA	NA	NA	NA	NA	NA	intron	BACE2	3.235	1
rs2837974	42602735	21	G	NA	NA	NA	NA	NA	NA	intron	BACE2	1.655	1
rs8127120	42619274	21	G	NA	NA	NA	NA	NA	NA	intron	BACE2	4.441	1
rs142329681	42619591	21	A	NA	NA	NA	NA	NA	NA	intron	BACE2	3.299	1
rs2837989	42620119	21	A	NA	NA	NA	NA	NA	NA	intron	BACE2	5.888	1
rs75303510	42620353	21	A	NA	NA	NA	NA	NA	NA	intron	BACE2	0.518	1
rs2837994	42624124	21	A	NA	NA	NA	NA	NA	NA	intron	BACE2	2.04	1
rs2837995	42624470	21	T	NA	NA	NA	NA	NA	NA	intron	BACE2	5.871	1
rs8134992	42624622	21	T	NA	NA	NA	NA	NA	NA	intron	BACE2	0.409	1
rs67525224	42627220	21	C	NA	NA	NA	NA	NA	NA	intron	BACE2	0.663	1
rs77395637	42627316	21	A	NA	NA	NA	NA	NA	NA	intron	BACE2	2.997	1
rs11702001	42627969	21	A	NA	NA	NA	NA	NA	NA	intron	BACE2	1.969	1
rs3787944	42629744	21	T	NA	NA	NA	NA	NA	NA	intron	BACE2	1.453	1

rs75748434	42630703	21	T	NA	NA	NA	NA	NA	NA	intron	BACE2	2.631	1
rs67837204	42632585	21	T	NA	NA	NA	NA	NA	NA	intron	BACE2	2.816	1
rs76228446	42632720	21	T	NA	NA	NA	NA	NA	NA	intron	BACE2	0.535	1
rs11701756	42632770	21	A	NA	NA	NA	NA	NA	NA	intron	BACE2	0.192	1
rs10483075	42633268	21	C	NA	NA	NA	NA	NA	NA	intron	BACE2	1.747	1
rs62219576	42633690	21	A	NA	NA	NA	NA	NA	NA	intron	BACE2	5.731	1
rs59836194	42634841	21	T	NA	NA	NA	NA	NA	NA	intron	BACE2	1.754	1
rs726980	42635676	21	A	NA	NA	NA	NA	NA	NA	intron	BACE2	0.938	1
rs74477222	42636501	21	T	NA	NA	NA	NA	NA	NA	intron	BACE2	1.786	1
rs7277675	42637222	21	T	NA	NA	NA	NA	NA	NA	intron	BACE2	0.433	1
rs58404061	42638931	21	A	NA	NA	NA	NA	NA	NA	intron	BACE2	1.743	1
rs3787945	42640022	21	T	NA	NA	NA	NA	NA	NA	intron	BACE2	1.743	1
rs75306103	42640410	21	A	NA	NA	NA	NA	NA	NA	intron	BACE2	0.758	1
rs58534150	42640903	21	T	NA	NA	NA	NA	NA	NA	intron	BACE2	0.04	1
rs68087522	42642096	21	G	NA	NA	NA	NA	NA	NA	intron	BACE2	5.155	1
rs77240271	42642405	21	A	NA	NA	NA	NA	NA	NA	intron	BACE2	2.167	1
rs58003932	42642825	21	G	NA	NA	NA	NA	NA	NA	intron	BACE2	5.921	1
rs62219579	42643610	21	C	NA	NA	NA	NA	NA	NA	intron	BACE2	1.035	1
rs75692138	42644452	21	A	NA	NA	NA	NA	NA	NA	intron	BACE2	3.888	1
rs75007172	42644515	21	A	NA	NA	NA	NA	NA	NA	intron	BACE2	5.027	1
rs58867243	42644663	21	A	NA	NA	NA	NA	NA	NA	intron	BACE2	11.57	1
rs34996007	42644885	21	T	NA	NA	NA	NA	NA	NA	intron	BACE2	9.399	1
rs11700718	42644982	21	C	NA	NA	NA	NA	NA	NA	intron	BACE2	2.431	1
rs61326441	42645938	21	A	NA	NA	NA	NA	NA	NA	intron	BACE2	1.147	1
rs62219580	42649211	21	G	NA	NA	NA	NA	NA	NA	intergenic	NA	2.516	1
rs73224110	42650666	21	A	NA	NA	NA	NA	NA	NA	intergenic	NA	0.295	1
rs11701157	42651480	21	A	NA	NA	NA	NA	NA	NA	intergenic	NA	0.139	1
rs111511447	42651495	21	A	NA	NA	NA	NA	NA	NA	intergenic	NA	0.654	1
rs3746889	42651946	21	C	NA	NA	NA	NA	NA	NA	intergenic	NA	0.381	1
rs11559045	42652831	21	T	NA	NA	NA	NA	NA	NA	intergenic	NA	0.346	1
rs117606823	42653122	21	G	NA	NA	NA	NA	NA	NA	intergenic	NA	4.62	1
rs73224118	42653246	21	T	NA	NA	NA	NA	NA	NA	intergenic	NA	9.325	1
rs1999331	42654497	21	A	NA	NA	NA	NA	NA	NA	intergenic	NA	0.914	1
rs75000682	42662479	21	A	NA	NA	NA	NA	NA	NA	intergenic	NA	2.753	1
rs73905312	42663603	21	A	NA	NA	NA	NA	NA	NA	intergenic	NA	12.5	1
rs73905316	42668059	21	T	NA	NA	NA	NA	NA	NA	intergenic	NA	0.853	1
rs67438118	42669465	21	A	NA	NA	NA	NA	NA	NA	intergenic	NA	0.185	1
rs76981906	42669740	21	A	NA	NA	NA	NA	NA	NA	intergenic	NA	3.008	1
rs7276719	42670165	21	A	NA	NA	NA	NA	NA	NA	intergenic	NA	6.32	1
rs146048697	42672680	21	T	NA	NA	NA	NA	NA	NA	intergenic	NA	6.555	1
rs4816716	42674021	21	A	NA	NA	NA	NA	NA	NA	intergenic	NA	0.667	1
rs6517662	42674022	21	A	NA	NA	NA	NA	NA	NA	intergenic	NA	0.726	1
rs75896199	42678194	21	G	NA	NA	NA	NA	NA	NA	intron	FAM3B	3.138	1

rs57347504	42678481	21	G	NA	NA	NA	NA	NA	NA	intron	FAM3B	0.926	1
rs68183718	42678791	21	T	NA	NA	NA	NA	NA	NA	intron	FAM3B	0.914	1

Supplementary Table 9

SNP	BP	CHR	A1	BETA	SE	L95	U95	STAT	P	LOCATION	GENESYMBOL	CADD	AdjP
rs2410415	42641402	21	C	-0.2738	0.1068	-0.4832	-0.06447	-2.564	0.01097	intron	BACE2	5.85	0.4717
rs8134468	42638685	21	A	-0.2719	0.1074	-0.4824	-0.0614	-2.532	0.01199	intron	BACE2	2.572	0.5156
rs2410404	42529908	21	T	-0.4214	0.2071	-0.8273	-0.01555	-2.035	0.0429	intergenic	NA	0.078	1
rs77270082	42531725	21	T	-0.4214	0.2071	-0.8273	-0.01555	-2.035	0.0429	intergenic	NA	3.295	1
rs74594237	42529023	21	A	-0.4195	0.2076	-0.8264	-0.01261	-2.021	0.04439	intergenic	NA	0.546	1
rs62217922	42532753	21	A	-0.4195	0.2076	-0.8264	-0.01261	-2.021	0.04439	intergenic	NA	0.959	1
rs3787933	42573589	21	G	0.2809	0.1832	-0.07819	0.6399	1.533	0.1265	intron	BACE2	0.916	1
rs12627075	42520443	21	C	-0.08881	0.06233	-0.211	0.03336	-1.425	0.1555	promoter	LINC00323	1.452	1
rs12627075	42520443	21	C	-0.08881	0.06233	-0.211	0.03336	-1.425	0.1555	intergenic	NA	1.452	1
rs9978431	42645737	21	T	0.08834	0.06534	-0.03973	0.2164	1.352	0.1777	intron	BACE2	4.656	1
rs2297271	42520327	21	G	-0.08244	0.0625	-0.2049	0.04005	-1.319	0.1884	promoter	LINC00323	1.617	1
rs2297271	42520327	21	G	-0.08244	0.0625	-0.2049	0.04005	-1.319	0.1884	intergenic	NA	1.617	1
rs2248555	42520337	21	G	-0.08244	0.0625	-0.2049	0.04005	-1.319	0.1884	promoter	LINC00323	3.998	1
rs2248555	42520337	21	G	-0.08244	0.0625	-0.2049	0.04005	-1.319	0.1884	intergenic	NA	3.998	1
rs2150425	42531759	21	A	0.07812	0.06122	-0.04187	0.1981	1.276	0.2032	intergenic	NA	7.144	1
rs11700917	42517222	21	T	0.2042	0.1627	-0.1146	0.5231	1.255	0.2106	intron	LINC00323	2.414	1
rs2016240	42530260	21	T	0.1935	0.1566	-0.1134	0.5003	1.236	0.2178	intergenic	NA	0.033	1
rs11700778	42517399	21	A	0.1971	0.1619	-0.1203	0.5144	1.217	0.2247	intron	LINC00323	1.427	1
rs11702409	42537012	21	G	0.1928	0.1606	-0.122	0.5077	1.201	0.2311	intergenic	NA	0.788	1
rs2898440	42517480	21	A	0.1943	0.1627	-0.1246	0.5132	1.194	0.2337	intron	LINC00323	1.485	1
rs914173	42517552	21	C	0.1943	0.1627	-0.1246	0.5132	1.194	0.2337	intron	LINC00323	1.058	1
rs914175	42517750	21	A	0.1943	0.1627	-0.1246	0.5132	1.194	0.2337	intron	LINC00323	2.443	1
rs7281814	42518444	21	G	0.1943	0.1627	-0.1246	0.5132	1.194	0.2337	intron	LINC00323	4.384	1
rs2837962	42518650	21	G	0.1943	0.1627	-0.1246	0.5132	1.194	0.2337	intron	LINC00323	9.818	1
rs6517653	42534253	21	A	0.1905	0.1601	-0.1234	0.5043	1.189	0.2354	intergenic	NA	0.477	1
rs2838002	42648767	21	C	0.1356	0.1141	-0.08806	0.3592	1.188	0.2359	intergenic	NA	2.123	1
rs2838003	42649357	21	C	0.1356	0.1141	-0.08806	0.3592	1.188	0.2359	intergenic	NA	3.146	1
rs2183588	42626882	21	A	0.0919	0.07865	-0.06226	0.2461	1.168	0.2438	intron	BACE2	1.744	1
rs9975388	42626362	21	C	0.09187	0.0788	-0.06258	0.2463	1.166	0.2448	intron	BACE2	4.067	1
rs960231	42622657	21	G	0.08934	0.07923	-0.06595	0.2446	1.128	0.2606	intron	BACE2	3.465	1
rs7277920	42575415	21	C	0.2344	0.2108	-0.1787	0.6475	1.112	0.2672	intron	BACE2	0.081	1
rs4816714	42574691	21	G	0.2333	0.2105	-0.1793	0.6458	1.108	0.2689	intron	BACE2	2.961	1
rs4818222	42574696	21	G	0.2333	0.2105	-0.1793	0.6458	1.108	0.2689	intron	BACE2	4.834	1
rs2007397	42516192	21	A	-0.06828	0.06249	-0.1908	0.05419	-1.093	0.2756	intron	LINC00323	0.221	1
rs28360666	42517239	21	T	-0.06612	0.06056	-0.1848	0.05258	-1.092	0.276	intron	LINC00323	0.431	1
rs2837961	42518599	21	C	-0.06486	0.06153	-0.1855	0.05575	-1.054	0.2929	intron	LINC00323	0.567	1
rs2210277	42536308	21	C	-0.06996	0.07193	-0.2109	0.07101	-0.9727	0.3317	intergenic	NA	1.445	1
rs2837964	42520646	21	A	0.09359	0.09827	-0.09901	0.2862	0.9524	0.3419	promoter	LINC00323	1.212	1
rs2837964	42520646	21	A	0.09359	0.09827	-0.09901	0.2862	0.9524	0.3419	intergenic	NA	1.212	1
rs1571732	42517559	21	G	0.09097	0.09698	-0.09911	0.2811	0.938	0.3492	intron	LINC00323	0.225	1

rs11088544	42517484	21	C	0.08996	0.09682	-0.09981	0.2797	0.9291	0.3537	intron	LINC00323	0.242	1
rs8133778	42642038	21	A	0.08916	0.09803	-0.103	0.2813	0.9095	0.364	intron	BACE2	6.941	1
rs2776342	42521213	21	C	0.08816	0.0972	-0.1023	0.2787	0.907	0.3653	promoter	LINC00323	2.557	1
rs2776342	42521213	21	C	0.08816	0.0972	-0.1023	0.2787	0.907	0.3653	intergenic	NA	2.557	1
rs2776343	42521267	21	A	0.08816	0.0972	-0.1023	0.2787	0.907	0.3653	promoter	LINC00323	0.128	1
rs2776343	42521267	21	A	0.08816	0.0972	-0.1023	0.2787	0.907	0.3653	intergenic	NA	0.128	1
rs2705572	42521161	21	G	0.08799	0.09704	-0.1022	0.2782	0.9067	0.3654	promoter	LINC00323	0.182	1
rs2705572	42521161	21	G	0.08799	0.09704	-0.1022	0.2782	0.9067	0.3654	intergenic	NA	0.182	1
rs2837999	42630531	21	A	0.08259	0.09109	-0.09594	0.2611	0.9067	0.3655	intron	BACE2	1.506	1
rs35939063	42584621	21	G	0.1653	0.1824	-0.1923	0.5229	0.9062	0.3657	intron	BACE2	4.171	1
rs13052926	42585088	21	G	0.1642	0.1827	-0.1939	0.5223	0.8987	0.3697	intron	BACE2	1.269	1
rs1041445	42515737	21	T	0.08904	0.09911	-0.1052	0.2833	0.8984	0.3699	intron	LINC00323	0.89	1
rs7276900	42532075	21	A	-0.06453	0.072	-0.2056	0.07659	-0.8962	0.371	intergenic	NA	6.65	1
rs6517655	42536246	21	C	-0.0634	0.07202	-0.2046	0.07776	-0.8803	0.3796	intergenic	NA	0.17	1
rs880370	42527504	21	A	0.04712	0.05528	-0.06122	0.1555	0.8525	0.3948	intergenic	NA	1.182	1
rs2837993	42620714	21	T	-0.05002	0.0589	-0.1655	0.06543	-0.8492	0.3966	intron	BACE2	0.306	1
rs2837996	42626706	21	T	0.06955	0.08304	-0.09319	0.2323	0.8376	0.4031	intron	BACE2	0.264	1
rs876817	42528499	21	C	-0.05894	0.07539	-0.2067	0.08882	-0.7818	0.4351	intergenic	NA	4.94	1
rs11702600	42524832	21	G	0.07271	0.09694	-0.1173	0.2627	0.75	0.4539	intergenic	NA	0.534	1
rs11701356	42522963	21	C	0.07348	0.09919	-0.1209	0.2679	0.7408	0.4595	intergenic	NA	1.832	1
rs10154102	42523427	21	G	0.07348	0.09919	-0.1209	0.2679	0.7408	0.4595	intergenic	NA	1.302	1
rs10154104	42523511	21	G	0.07348	0.09919	-0.1209	0.2679	0.7408	0.4595	intergenic	NA	1.873	1
rs11701851	42523852	21	T	0.07296	0.0993	-0.1217	0.2676	0.7348	0.4631	intergenic	NA	0.831	1
rs11702163	42524513	21	C	0.07296	0.0993	-0.1217	0.2676	0.7348	0.4631	intergenic	NA	1.858	1
rs11702590	42524628	21	A	0.07296	0.0993	-0.1217	0.2676	0.7348	0.4631	intergenic	NA	6.287	1
rs11702752	42524741	21	C	0.07296	0.0993	-0.1217	0.2676	0.7348	0.4631	intergenic	NA	0.789	1
rs11702604	42524870	21	T	0.07296	0.0993	-0.1217	0.2676	0.7348	0.4631	intergenic	NA	4.253	1
rs10154234	42525007	21	A	0.07296	0.0993	-0.1217	0.2676	0.7348	0.4631	intergenic	NA	3.608	1
rs9305724	42525185	21	G	0.07296	0.0993	-0.1217	0.2676	0.7348	0.4631	intergenic	NA	1.61	1
rs9305725	42525257	21	C	0.07296	0.0993	-0.1217	0.2676	0.7348	0.4631	intergenic	NA	1.78	1
rs9305726	42525272	21	A	0.07296	0.0993	-0.1217	0.2676	0.7348	0.4631	intergenic	NA	0.259	1
rs9305727	42525411	21	G	0.07296	0.0993	-0.1217	0.2676	0.7348	0.4631	intergenic	NA	0.379	1
rs9305728	42525448	21	A	0.07296	0.0993	-0.1217	0.2676	0.7348	0.4631	intergenic	NA	8.162	1
rs13049454	42526520	21	A	0.07296	0.0993	-0.1217	0.2676	0.7348	0.4631	intergenic	NA	2.152	1
rs7278856	42598887	21	G	-0.1114	0.1564	-0.4179	0.195	-0.7127	0.4767	intron	BACE2	4.367	1
rs35335714	42517341	21	C	0.07094	0.1	-0.1251	0.2669	0.7094	0.4788	intron	LINC00323	0.889	1
rs2837971	42601214	21	A	-0.1134	0.1627	-0.4322	0.2054	-0.6972	0.4864	intron	BACE2	0.629	1
rs11701197	42522788	21	A	0.06874	0.09949	-0.1263	0.2637	0.6909	0.4903	intergenic	NA	2.388	1
rs1077117	42599364	21	G	-0.1059	0.1571	-0.4138	0.2019	-0.6744	0.5007	intron	BACE2	4.443	1
rs9980363	42520134	21	C	0.06647	0.09995	-0.1294	0.2624	0.6651	0.5066	promoter	LINC00323	3.326	1
rs9980363	42520134	21	C	0.06647	0.09995	-0.1294	0.2624	0.6651	0.5066	intergenic	NA	3.326	1
rs9982271	42519389	21	T	0.06192	0.0965	-0.1272	0.2511	0.6416	0.5217	intron	LINC00323	5.274	1
rs11701489	42517643	21	A	0.06147	0.09899	-0.1326	0.2555	0.6209	0.5352	intron	LINC00323	0.265	1

rs6517659	42641982	21	T	-0.1041	0.1683	-0.434	0.2259	-0.6183	0.537	intron	BACE2	2.702	1
rs9984070	42516327	21	T	0.06031	0.09927	-0.1343	0.2549	0.6076	0.544	intron	LINC00323	1.813	1
rs9974272	42521711	21	G	0.06943	0.1184	-0.1626	0.3015	0.5864	0.5582	promoter	LINC00323	1.91	1
rs9974272	42521711	21	G	0.06943	0.1184	-0.1626	0.3015	0.5864	0.5582	intergenic	NA	1.91	1
rs11700578	42522350	21	C	-0.04437	0.07606	-0.1934	0.1047	-0.5834	0.5602	intergenic	NA	1.044	1
rs11700757	42522447	21	T	-0.04437	0.07606	-0.1934	0.1047	-0.5834	0.5602	intergenic	NA	0.072	1
rs11700599	42522499	21	C	-0.04437	0.07606	-0.1934	0.1047	-0.5834	0.5602	intergenic	NA	8.106	1
rs11701114	42522656	21	A	-0.04437	0.07606	-0.1934	0.1047	-0.5834	0.5602	intergenic	NA	0.019	1
rs2012050	42651562	21	T	0.04357	0.07821	-0.1097	0.1969	0.557	0.578	intergenic	NA	4.184	1
rs12483323	42643845	21	T	-0.09289	0.1683	-0.4228	0.237	-0.5518	0.5816	intron	BACE2	2.526	1
rs914174	42517678	21	A	0.04453	0.08532	-0.1227	0.2117	0.522	0.6021	intron	LINC00323	1.085	1
rs9983938	42516357	21	G	0.04327	0.08562	-0.1245	0.2111	0.5054	0.6138	intron	LINC00323	2.264	1
rs12482462	42626851	21	T	-0.02774	0.05828	-0.142	0.08648	-0.476	0.6345	intron	BACE2	11.02	1
rs1571733	42518034	21	A	-0.08606	0.1827	-0.4442	0.2721	-0.471	0.6381	intron	LINC00323	3.33	1
rs2837990	42620149	21	A	-0.07493	0.1591	-0.3868	0.237	-0.4709	0.6381	intron	BACE2	4.605	1
rs2837991	42620160	21	A	-0.07493	0.1591	-0.3868	0.237	-0.4709	0.6381	intron	BACE2	3.068	1
rs9981553	42620680	21	T	-0.02651	0.05831	-0.1408	0.08778	-0.4546	0.6498	intron	BACE2	2.454	1
rs11909506	42678742	21	G	0.0321	0.0772	-0.1192	0.1834	0.4158	0.6779	intron	FAM3B	0.63	1
rs7279273	42664100	21	C	0.02569	0.06182	-0.09548	0.1469	0.4156	0.6781	intergenic	NA	3.185	1
rs9975636	42531333	21	A	0.03718	0.08993	-0.1391	0.2134	0.4134	0.6797	intergenic	NA	1.541	1
rs8134160	42541202	21	T	0.03177	0.08024	-0.1255	0.189	0.3959	0.6925	intron	BACE2	4.229	1
rs8127156	42543403	21	G	0.03079	0.07981	-0.1256	0.1872	0.3858	0.7	intron	BACE2	4.064	1
rs9980188	42664366	21	G	0.02286	0.06159	-0.09785	0.1436	0.3711	0.7109	intergenic	NA	0.133	1
rs13049769	42664689	21	C	0.02286	0.06159	-0.09785	0.1436	0.3711	0.7109	intergenic	NA	0.243	1
rs28654619	42536126	21	C	0.03238	0.09009	-0.1442	0.2089	0.3594	0.7196	intergenic	NA	3.37	1
rs6517654	42534328	21	C	0.02983	0.08494	-0.1366	0.1963	0.3512	0.7257	intergenic	NA	0.441	1
rs62217923	42539293	21	G	0.02473	0.07814	-0.1284	0.1779	0.3164	0.7519	promoter	MIR3197	7.924	1
rs62217923	42539293	21	G	0.02473	0.07814	-0.1284	0.1779	0.3164	0.7519	promoter	BACE2	7.924	1
rs62217923	42539293	21	G	0.02473	0.07814	-0.1284	0.1779	0.3164	0.7519	intergenic	NA	7.924	1
rs55853145	42524465	21	A	-0.03433	0.1133	-0.2565	0.1878	-0.3029	0.7622	intergenic	NA	3.721	1
rs67757006	42525663	21	A	-0.03433	0.1133	-0.2565	0.1878	-0.3029	0.7622	intergenic	NA	0.396	1
rs57911156	42522469	21	C	-0.03424	0.1136	-0.2568	0.1884	-0.3015	0.7633	intergenic	NA	1.935	1
rs9976426	42641913	21	T	-0.01468	0.06021	-0.1327	0.1033	-0.2439	0.8075	intron	BACE2	1.811	1
rs9305733	42642699	21	C	-0.01468	0.06021	-0.1327	0.1033	-0.2439	0.8075	intron	BACE2	1.263	1
rs10775668	42678029	21	T	0.0214	0.08988	-0.1548	0.1976	0.2381	0.812	intron	FAM3B	5.034	1
rs9976125	42678988	21	G	0.0211	0.08977	-0.1549	0.1971	0.235	0.8144	intron	FAM3B	0.473	1
rs9976433	42679010	21	A	0.0211	0.08977	-0.1549	0.1971	0.235	0.8144	intron	FAM3B	3.661	1
rs9976216	42679037	21	G	0.0211	0.08977	-0.1549	0.1971	0.235	0.8144	intron	FAM3B	1.672	1
rs9976217	42679042	21	G	0.0211	0.08977	-0.1549	0.1971	0.235	0.8144	intron	FAM3B	0.533	1
rs9976965	42679090	21	C	0.0211	0.08977	-0.1549	0.1971	0.235	0.8144	intron	FAM3B	0.561	1
rs62217917	42527354	21	A	-0.02314	0.1086	-0.2359	0.1897	-0.2131	0.8314	intergenic	NA	3.01	1
rs12482242	42648821	21	G	-0.01235	0.06003	-0.13	0.1053	-0.2058	0.8372	intergenic	NA	5.968	1
rs2838004	42650654	21	G	-0.01235	0.06003	-0.13	0.1053	-0.2058	0.8372	intergenic	NA	11.16	1

rs960230	42622479	21	A	0.02332	0.1138	-0.1997	0.2464	0.2049	0.8378	intron	BACE2	0.443	1
rs2838001	42646992	21	C	-0.03185	0.1603	-0.346	0.2823	-0.1987	0.8426	intron	BACE2	0.276	1
rs2838000	42646518	21	A	-0.03162	0.1604	-0.3459	0.2827	-0.1972	0.8439	intron	BACE2	0.091	1
rs62217915	42519037	21	C	-0.04107	0.2102	-0.4531	0.371	-0.1953	0.8453	intron	LINC00323	1.443	1
rs1810868	42651481	21	A	-0.00864	0.06177	-0.1297	0.1124	-0.1398	0.8889	intergenic	NA	0.102	1
rs6517657	42584141	21	T	0.01279	0.15	-0.2811	0.3067	0.08532	0.9321	intron	BACE2	1.966	1
rs34019132	42583154	21	T	0.01199	0.1501	-0.2822	0.3062	0.07991	0.9364	intron	BACE2	1.863	1
rs6517656	42583738	21	A	0.01206	0.1514	-0.2847	0.3088	0.07966	0.9366	intron	BACE2	15.65	1
rs28360503	42583804	21	C	0.01164	0.1502	-0.2827	0.306	0.07753	0.9383	intron	BACE2	1.192	1
rs9808711	42642642	21	A	0.008813	0.1619	-0.3085	0.3261	0.05444	0.9566	intron	BACE2	2.893	1
rs4818229	42643968	21	T	0.008813	0.1619	-0.3085	0.3261	0.05444	0.9566	intron	BACE2	3.003	1
rs4818228	42643772	21	A	0.00704	0.1627	-0.3118	0.3258	0.04328	0.9655	intron	BACE2	2.695	1
rs7279020	42534031	21	T	-0.00177	0.06029	-0.1199	0.1164	-0.02931	0.9766	intergenic	NA	1.664	1
rs914176	42528192	21	G	0.001635	0.05997	-0.1159	0.1192	0.02726	0.9783	intergenic	NA	0.126	1
rs13048452	42532909	21	G	-0.00046	0.06042	-0.1189	0.118	-0.00755	0.994	intergenic	NA	1.938	1
rs11088546	42532977	21	A	-0.00046	0.06042	-0.1189	0.118	-0.00755	0.994	intergenic	NA	2.54	1
rs57576269	42516902	21	A	NA	NA	NA	NA	NA	NA	intron	LINC00323	2.078	1
rs73362453	42519604	21	T	NA	NA	NA	NA	NA	NA	intron	LINC00323	0.094	1
rs73364465	42577778	21	G	NA	NA	NA	NA	NA	NA	intron	BACE2	0.367	1
rs75762331	42581079	21	A	NA	NA	NA	NA	NA	NA	intron	BACE2	1.284	1
rs2837969	42588377	21	T	NA	NA	NA	NA	NA	NA	intron	BACE2	1.183	1
rs10451740	42588731	21	C	NA	NA	NA	NA	NA	NA	intron	BACE2	1.848	1
rs80303768	42594678	21	A	NA	NA	NA	NA	NA	NA	intron	BACE2	4.279	1
rs28629220	42597387	21	C	NA	NA	NA	NA	NA	NA	intron	BACE2	3.563	1
rs9305731	42598396	21	G	NA	NA	NA	NA	NA	NA	intron	BACE2	2.492	1
rs17000688	42599218	21	A	NA	NA	NA	NA	NA	NA	intron	BACE2	4.117	1
rs12627301	42599787	21	C	NA	NA	NA	NA	NA	NA	intron	BACE2	0.684	1
rs9982181	42599843	21	T	NA	NA	NA	NA	NA	NA	intron	BACE2	3.303	1
rs59760478	42600934	21	A	NA	NA	NA	NA	NA	NA	intron	BACE2	1.333	1
rs2837972	42601741	21	G	NA	NA	NA	NA	NA	NA	intron	BACE2	1.213	1
rs2837973	42602088	21	G	NA	NA	NA	NA	NA	NA	intron	BACE2	3.235	1
rs2837974	42602735	21	G	NA	NA	NA	NA	NA	NA	intron	BACE2	1.655	1
rs8127120	42619274	21	G	NA	NA	NA	NA	NA	NA	intron	BACE2	4.441	1
rs142329681	42619591	21	A	NA	NA	NA	NA	NA	NA	intron	BACE2	3.299	1
rs2837989	42620119	21	A	NA	NA	NA	NA	NA	NA	intron	BACE2	5.888	1
rs75303510	42620353	21	A	NA	NA	NA	NA	NA	NA	intron	BACE2	0.518	1
rs2837994	42624124	21	A	NA	NA	NA	NA	NA	NA	intron	BACE2	2.04	1
rs2837995	42624470	21	T	NA	NA	NA	NA	NA	NA	intron	BACE2	5.871	1
rs8134992	42624622	21	T	NA	NA	NA	NA	NA	NA	intron	BACE2	0.409	1
rs67525224	42627220	21	C	NA	NA	NA	NA	NA	NA	intron	BACE2	0.663	1
rs77395637	42627316	21	A	NA	NA	NA	NA	NA	NA	intron	BACE2	2.997	1
rs11702001	42627969	21	A	NA	NA	NA	NA	NA	NA	intron	BACE2	1.969	1
rs3787944	42629744	21	T	NA	NA	NA	NA	NA	NA	intron	BACE2	1.453	1

rs75748434	42630703	21	T	NA	NA	NA	NA	NA	NA	intron	BACE2	2.631	1
rs67837204	42632585	21	T	NA	NA	NA	NA	NA	NA	intron	BACE2	2.816	1
rs76228446	42632720	21	T	NA	NA	NA	NA	NA	NA	intron	BACE2	0.535	1
rs11701756	42632770	21	A	NA	NA	NA	NA	NA	NA	intron	BACE2	0.192	1
rs10483075	42633268	21	C	NA	NA	NA	NA	NA	NA	intron	BACE2	1.747	1
rs62219576	42633690	21	A	NA	NA	NA	NA	NA	NA	intron	BACE2	5.731	1
rs59836194	42634841	21	T	NA	NA	NA	NA	NA	NA	intron	BACE2	1.754	1
rs726980	42635676	21	A	NA	NA	NA	NA	NA	NA	intron	BACE2	0.938	1
rs74477222	42636501	21	T	NA	NA	NA	NA	NA	NA	intron	BACE2	1.786	1
rs7277675	42637222	21	T	NA	NA	NA	NA	NA	NA	intron	BACE2	0.433	1
rs58404061	42638931	21	A	NA	NA	NA	NA	NA	NA	intron	BACE2	1.743	1
rs3787945	42640022	21	T	NA	NA	NA	NA	NA	NA	intron	BACE2	1.743	1
rs75306103	42640410	21	A	NA	NA	NA	NA	NA	NA	intron	BACE2	0.758	1
rs58534150	42640903	21	T	NA	NA	NA	NA	NA	NA	intron	BACE2	0.04	1
rs68087522	42642096	21	G	NA	NA	NA	NA	NA	NA	intron	BACE2	5.155	1
rs77240271	42642405	21	A	NA	NA	NA	NA	NA	NA	intron	BACE2	2.167	1
rs58003932	42642825	21	G	NA	NA	NA	NA	NA	NA	intron	BACE2	5.921	1
rs62219579	42643610	21	C	NA	NA	NA	NA	NA	NA	intron	BACE2	1.035	1
rs75692138	42644452	21	A	NA	NA	NA	NA	NA	NA	intron	BACE2	3.888	1
rs75007172	42644515	21	A	NA	NA	NA	NA	NA	NA	intron	BACE2	5.027	1
rs58867243	42644663	21	A	NA	NA	NA	NA	NA	NA	intron	BACE2	11.57	1
rs34996007	42644885	21	T	NA	NA	NA	NA	NA	NA	intron	BACE2	9.399	1
rs11700718	42644982	21	C	NA	NA	NA	NA	NA	NA	intron	BACE2	2.431	1
rs61326441	42645938	21	A	NA	NA	NA	NA	NA	NA	intron	BACE2	1.147	1
rs62219580	42649211	21	G	NA	NA	NA	NA	NA	NA	intergenic	NA	2.516	1
rs73224110	42650666	21	A	NA	NA	NA	NA	NA	NA	intergenic	NA	0.295	1
rs11701157	42651480	21	A	NA	NA	NA	NA	NA	NA	intergenic	NA	0.139	1
rs111511447	42651495	21	A	NA	NA	NA	NA	NA	NA	intergenic	NA	0.654	1
rs3746889	42651946	21	C	NA	NA	NA	NA	NA	NA	intergenic	NA	0.381	1
rs11559045	42652831	21	T	NA	NA	NA	NA	NA	NA	intergenic	NA	0.346	1
rs117606823	42653122	21	G	NA	NA	NA	NA	NA	NA	intergenic	NA	4.62	1
rs73224118	42653246	21	T	NA	NA	NA	NA	NA	NA	intergenic	NA	9.325	1
rs1999331	42654497	21	A	NA	NA	NA	NA	NA	NA	intergenic	NA	0.914	1
rs75000682	42662479	21	A	NA	NA	NA	NA	NA	NA	intergenic	NA	2.753	1
rs73905312	42663603	21	A	NA	NA	NA	NA	NA	NA	intergenic	NA	12.5	1
rs73905316	42668059	21	T	NA	NA	NA	NA	NA	NA	intergenic	NA	0.853	1
rs67438118	42669465	21	A	NA	NA	NA	NA	NA	NA	intergenic	NA	0.185	1
rs76981906	42669740	21	A	NA	NA	NA	NA	NA	NA	intergenic	NA	3.008	1
rs7276719	42670165	21	A	NA	NA	NA	NA	NA	NA	intergenic	NA	6.32	1
rs146048697	42672680	21	T	NA	NA	NA	NA	NA	NA	intergenic	NA	6.555	1
rs4816716	42674021	21	A	NA	NA	NA	NA	NA	NA	intergenic	NA	0.667	1
rs6517662	42674022	21	A	NA	NA	NA	NA	NA	NA	intergenic	NA	0.726	1
rs75896199	42678194	21	G	NA	NA	NA	NA	NA	NA	intron	FAM3B	3.138	1

rs57347504	42678481	21	G	NA	NA	NA	NA	NA	NA	intron	FAM3B	0.926	1
rs68183718	42678791	21	T	NA	NA	NA	NA	NA	NA	intron	FAM3B	0.914	1
